# Supplementary material for: Preventing PTSD, depression and associated health problems in student paramedics: protocol for PREVENT-PTSD, a randomised controlled trial of supported online cognitive training for resilience versus alternative online training and standard practice
Source: BMJ Open. 2018 Dec 31;8(12):e022292. doi: 10.1136/bmjopen-2018-022292 (PMC6318590; doi:10.1136/bmjopen-2018-022292)
Supplement: Supplementary data [file bmjopen-2018-022292supp001.pdf]

Appendix 1. World Health Organization Trial Registration Data Set.

DATA CATEGORY	INFORMATION
Primary registry and trial identifying number	Registry: ISRCTN. Identifying number: ISRCTN16493616.
Date of registration in primary registry	9 October, 2017
Secondary identifying numbers	Protocol/serial number: PREVENT-PTSD/Protocol V1.
Source(s) of monetary or material support	MQ: Transforming Mental Health
Primary sponsor	MQ: Transforming Mental Health
Secondary sponsor(s)	University of Oxford
Contact for public queries	*JW* +44(0)1865 618612, jennifer.wild@psy.ox.ac.uk
Contact for scientific queries	*JW* +44(0)1865 618612, jennifer.wild@psy.ox.ac.uk
Public title	PREVENT-PTSD
Scientific title	Preventing PTSD, depression, and associated health problems in student paramedics: Protocol for PREVENT-PTSD, a randomised controlled trial of supported online cognitive training for resilience versus alternative online training and standard practice
Countries of recruitment	England
Health condition(s) or problem(s) studied	Post-traumatic stress disorder (PTSD), major depression (MD), resilience, adverse health behaviours (sleep problems, weight gain and smoking), hormone and immune function, utilization of health services and productivity loss.
Intervention(s)	Active comparator: Resilience intervention focusing on modifying rumination and cognitive appraisals<br>Control comparator: Online information and advice about stress, sleep problems, anger, depression PTSD and mindfulness<br>Standard practice comparator: Training as usual
Key inclusion and exclusion criteria	Ages eligible for study: ≥18 years<br>Sexes eligible for study: both<br>Accepts healthy volunteers: yes<br>Inclusion criteria: student paramedics (≥ 18 years) who do not currently have depression or PTSD needing treatment and who are not actively suicidal.<br>Exclusion criteria: student paramedics who meet criteria for depression or PTSD and whose lives are significantly impacted by their symptoms, thereby needing treatment; student paramedics who express suicidal ideation and intent.
Study type	Interventional<br>Allocation: randomised<br>Intervention model: parallel assignment<br>Masking: assessors blinded to all assessments<br>Primary purpose: prevention<br>Phase I
Date of first enrolment	October 2017
Target sample size	570

Recruitment status	Recruiting
Primary outcome(s)	Diagnoses of PTSD and MD assessed by an independent assessor using the Structured Clinical Interview for DSM-5
Key secondary outcomes	Mental Health: resilience, rumination, symptoms of anxiety, and sleep problems, psychological distress, wellbeing<br><br>Immune and Endocrine Function: cortisol awakening response and plasma levels of C-reactive protein<br><br>Physical health behaviours: smoking, weight gain, alcohol use.<br><br>Health Economics: utilisation of health services, episodes of PTSD and MD, absenteeism and presenteeism
