# Supplementary material for: Preventing PTSD, depression and associated health problems in student paramedics: protocol for PREVENT-PTSD, a randomised controlled trial of supported online cognitive training for resilience versus alternative online training and standard practice
Source: BMJ Open. 2018 Dec 31;8(12):e022292. doi: 10.1136/bmjopen-2018-022292 (PMC6318590; doi:10.1136/bmjopen-2018-022292)
Supplement: Supplementary data [file bmjopen-2018-022292supp003.pdf]

Oxford Centre for Anxiety Disorders and Trauma
The Old Rectory
Paradise Square, Oxford
OX1 1TW

Dr Jennifer Wild
Jennifer.wild@psy.ox.ac.uk Tel: 01865 618 612
Gabriella Tyson (Research Assistant)
Oxford telephone number: 01865 618 610
Oxford email address: gabriella.tyson@psy.ox.ac.uk

A study of resilience training for student paramedics

PARTICIPANT INFORMATION SHEET

Ethics Approval Reference: R44116/RE001

1. ***Background and aims of the study***

Research indicates that paramedics carry an increased risk for depression and a severe stress condition called posttraumatic stress disorder (PTSD) due to the nature of their work. Our past research identified early predictors of these problems in student paramedics. We have now developed a training programme that aims to prevent these problems from developing by modifying the predictors linked to their onset. The study hopes to answer the following questions:

Do student paramedics benefit from resilience training?
Which intervention, if any, best helps student paramedics?
Are the interventions associated with improvements in physical health?

This study is funded by MQ. The courses are free and will be delivered online with the support of a wellbeing coach.

2. ***Why have I been invited to take part?***

You have been invited to take part in the study because you are a student paramedic between the ages of 18 and 65 years.

The inclusion criteria are students who are training to be paramedics and who are in years 1, 2 or 3 of their paramedic programme.

The exclusion criteria are students who score in the clinical range on screening measures of post-traumatic stress and depression and would also benefit from treatment since psychological treatment is likely to be more helpful.

3. ***Do I have to take part?***

No. You can ask questions about the study before deciding whether or not to participate. If you do agree to participate, you may withdraw yourself and your data from the study at any time, without giving a reason and without penalty, by advising the researchers of this decision.

4. ***What will happen in the study?***

If you are happy to take part in the study, you will be asked to fill in two short questionnaires about depression and anxiety. You will not be able to take part if these questionnaires suggest that you may have

one of these problems and would also benefit from treatment. If this is the case, the researcher will talk with you and give you suggestions about what may be helpful. This could be a visit to your GP or accessing other local services or both. The screening questionnaires will be destroyed after use.

You will be able to take part if the questionnaires suggest that you do not have depression or post-traumatic stress.

You will be invited to complete a longer set of questionnaires that measure levels of wellbeing, resilience and stress and to answer a few questions about stress symptoms over the telephone with our research assistant at a time that is convenient to you. Once you have completed these, you will be randomly allocated to one of the two internet-based courses which will start within a few weeks or to standard practice, which means you would receive an internet-based course at the end of two years. 70% of the people in the study will be able to start the course right away. The remaining 30% will be offered the course after two years. **The decision about which intervention you will receive will be made by chance.**

You will be invited to give a blood sample (1 teaspoon) before the course (or standard practice), immediately after, 12 and 24 months post course (or standard practice). Blood samples will be taken by trained staff. Samples will be analysed for a marker of inflammation called C-reactive protein. No cellular constituents will be stored or analysed.

You will also be invited to take 6 samples of your saliva (to measure cortisol, a stress hormone) upon awakening, 15, 30 and 60 minutes after awakening, and at 12 noon and 8 pm. Full instructions will be given on how to do this in your home. You will be provided with a Royal Mail Safebox to securely post the samples to the University of Surrey where they will be analysed.

The main phase of the course is 6 weeks. If you are allocated to either of the internet-based courses you will work through the internet programme modules in the comfort of your home with support from a wellbeing coach via SMS or email, depending on your preference. The internet programme will require you to dedicate up to an hour a week in the first 6 weeks of the course, and after that you will receive regular reminders and top up exercises.

Over the course of the interventions, we will ask you to complete questionnaires at five time points: before the intervention, after the intervention, six, 12 and 24 months after the intervention. The questionnaires take 20 minutes to complete at all time points except at 6 months post-intervention, when they will take just 10 minutes to complete. Therefore 105 minutes in the first year and 80 minutes in the second year would be required to complete questionnaires and telephone interviews. The timing of visits and what would be done at each visit is below:

Baseline
At home – two online questionnaires (10 minutes)

1 to 7 days later
At home – online questionnaires (20 minutes) + telephone interview (15 minutes) + saliva samples (10 minutes)
Visit 1 at university – Blood sample with our phlebotomist

6 weeks later
At home – online questionnaires (20 minutes) + saliva samples (10 minutes) + telephone interview (10 minutes)
Visit 2 at university - Blood sample with our phlebotomist

24 weeks later
At home – online questionnaires (10 minutes)

12 months later
At home – online questionnaires (20 minutes) + saliva samples (10 minutes) + telephone interview (15

minutes)
Visit 3 at university - Blood sample with our phlebotomist

24 months later
At home – online questionnaires (20 minutes) + saliva samples (10 minutes) + telephone interview (15 minutes)
Visit 4 at university - Blood sample with our phlebotomist

The telephone interviews will be audio-recorded. This is so that at a later date an independent assessor can rate a random sample of audio-recordings to ensure that the interviews have followed the study protocol. This process refers to quality assurance. The audio files are confidential and will be securely transferred to the desktop of the principal researcher at the University of Oxford. All audio files will be deleted within 7 years of the study ending.

5. ***Are there any potential risks in taking part?***

There are no risks associated with completing the questionnaires or the interventions or receiving standard practice or taking saliva samples. There are common risks associated with taking blood. It can be uncomfortable and result in fainting, localised pain, or bruising.

6. ***Are there any benefits in taking part?***

There are significant benefits from taking part in this research. Your participation could lead to improvements in your resilience and mental wellbeing and your participation will help us in evaluating the resilience intervention, which will guide improvements to the course before it is made nationally available.

7. ***Expenses and payments***

You will receive £30 for participation at the end of the study.

8. ***What will happen to any samples I give?***

Your saliva samples will be analysed for levels of a stress hormone called cortisol and destroyed immediately following analysis by incineration. We will process your blood samples within 24 hours of collection. Serum, which contains no cellular constituents, will be stored and analysed for an immune marker called C-reactive protein. Serum samples will be destroyed by incineration at the end of the study.

9. ***What happens to the data provided?***

Your name will be removed from your questionnaires and the anonymised research data will be stored on a password protected computer at the Oxford Centre for Anxiety Disorders and Trauma, Department of Experimental Psychology, University of Oxford. Anonymised saliva samples identified by a unique code will be analysed by the Biochemistry and Physiology Laboratory at the University of Surrey and immediately destroyed.

The Biochemistry and Physiology Laboratory at the University of Surrey will also process anonymised blood samples within 24 hours of collection, which will be analysed for a marker of inflammation called C-reactive protein. No cellular constituents will be saved or stored.

All information you provide will be strictly confidential. However, responsible members of the University of Oxford or King's College London may be given access to data for monitoring and/or audit or to suggest that specific analyses are carried out at the end of the study. We are collaborating with an expert in biological stress responses (Professor Carmine Pariante, King's College London), an expert in immune function (Professor Andrea Danese, King's College London) and an expert in health economics (Apostolos

Tsiachristas, University of Oxford). Our collaborators may suggest that we conduct specific statistical analyses at the end of the study.

In order to support transparency in research, some journals request that the aggregated anonymised data collected during a study are deposited within the UK data archive. If the journal with which we publish requires this, then the anonymised data would be submitted to the repository one year after the study is completed. Please be assured that only numerical data relating to the study outcomes (no personal identifying information whatsoever) would be held in the repository. We may also share the aggregated anonymised data with responsible researchers with an interest in resilience interventions.

To access the online courses, you will need to enter your unique code. Your responses in the modules you complete online will be linked to this code and stored anonymously on a password-protected database only accessible by the Principal Investigator and the research assistant. The responses linked to each code may be analysed.

Personal data relating to gender and ethnicity will be coded and then anonymised by linking it to your participant code rather than your name. Data will be stored on a password protected database on the Principal Investigator's computer. Your consent form will be stored in a locked filing cabinet in the Principal Investigator's office for 7 years and then destroyed.

10. Will the research be published?

The results from this study may be published within the next 7 years. You will not be personally identified in any literature and can obtain a copy of any publications from the contact numbers below.

11. Who has reviewed this study?

This study has been reviewed by, and received ethics clearance through, the University of Oxford Central University Research Ethics Committee.

12. *Who do I contact if I have a concern about the study or I wish to complain?*

If you have a concern about any aspect of this study, please speak to the Gabriella Tyson [01865 618 610] or Dr Jennifer Wild [01865 618 612], who will do their best to answer your query. The researcher should acknowledge your concern within 10 working days and give you an indication of how they intend to deal with it. If you remain unhappy or wish to make a formal complaint, please contact the relevant chair of the Research Ethics Committee at the University of Oxford who will seek to resolve the matter in a reasonably expeditious manner:

Chair, **Medical Sciences Inter-Divisional Research Ethics Committee**; Email: ethics@medsci.ox.ac.uk;
Address: Research Services, University of Oxford, Wellington Square, Oxford OX1 2JD

13. Further Information and Contact Details

If you would like to discuss the research with someone beforehand (or if you have questions afterwards), please contact:

Dr Jennifer Wild
Oxford Centre for Anxiety Disorders and Trauma
The Old Rectory
Paradise Square

Oxford
OX1 1TW
Tel: 01865 618 612
Email: Jennifer.wild@psy.ox.ac.uk

Oxford Centre for Anxiety Disorders and Trauma
The Old Rectory
Paradise Square, Oxford
OX1 1TW

Dr Jennifer Wild
Jennifer.wild@psy.ox.ac.uk Tel: 01865 618 612
Gabriella Tyson (Research Assistant)
Oxford telephone number: 01865 618 610
Oxford email address: gabriella.tyson@psy.ox.ac.ukx.ac.uk Tel: 01865 618 612

PARTICIPANT CONSENT FORM

CUREC Approval Reference:

A Study of Resilience Training for Student Paramedics

Purpose of Study: To evaluate a new resilience intervention developed for student paramedics

		Please initial each box
1	I confirm that I have read and understand the information sheet version_____dated _________________ for the above study. I have had the opportunity to consider the information, ask questions and have had these answered satisfactorily.	
2	I understand that my participation is voluntary and that I am free to withdraw at any time, without giving any reason, and without any adverse consequences or academic penalty.	
3	I understand that research data collected during the study may be looked at by designated individuals from the University of Oxford where it is relevant to my taking part in this study. I give permission for these individuals to access my data.	
4	I understand that this project has been reviewed by, and received ethics clearance through, the University of Oxford Central University Research Ethics Committee.	
5	I understand who will have access to personal data provided, how the data will be stored and what will happen to the data at the end of the project.	
6	I give permission for the aggregated anonymised data to be shared with the UK data archive and other responsible researchers.	
7.	I consent to my telephone interviews with the research assistant being audio-recorded for quality assurance purposes.	
8	I understand how this research will be written up and published.	
9	I understand how to raise a concern or make a complaint.	
**10**	I understand that a blood and saliva will be taken during the study and that these samples will be tested for C-reactive protein and cortisol respectively. I understand that the samples will be destroyed after completion of this test or if I withdraw my consent for the test.	

11 I consider these samples a gift to the University of Oxford and I understand I will not gain any direct personal benefit from this. ☐

12 I agree to take part in the above study. ☐

______________________	____________	____________________________
Name of Participant	Date	Signature

______________________	____________	____________________________
Name of person taking consent	Date	Signature
