# Supplementary material for: Preventing PTSD, depression and associated health problems in student paramedics: protocol for PREVENT-PTSD, a randomised controlled trial of supported online cognitive training for resilience versus alternative online training and standard practice
Source: BMJ Open. 2018 Dec 31;8(12):e022292. doi: 10.1136/bmjopen-2018-022292 (PMC6318590; doi:10.1136/bmjopen-2018-022292)
Supplement: Author's manuscript [file bmjopen-2018-022292.draft_revisions.pdf]

BMJ Open

BMJ Open is committed to open peer review. As part of this commitment we make the peer review history of every article we publish publicly available.

When an article is published we post the peer reviewers' comments and the authors' responses online. We also post the versions of the paper that were used during peer review. These are the versions that the peer review comments apply to.

The versions of the paper that follow are the versions that were submitted during the peer review process. They are not the versions of record or the final published versions. They should not be cited or distributed as the published version of this manuscript.

BMJ Open is an open access journal and the full, final, typeset and author-corrected version of record of the manuscript is available on our site with no access controls, subscription charges or pay-per-view fees (http://bmjopen.bmj.com).

If you have any questions on BMJ Open's open peer review process please email editorial.bmjopen@bmj.com

Preventing PTSD, depression, and associated health problems in student paramedics: Protocol for PREVENT-PTSD, a randomised controlled trial of supported online cognitive training for resilience versus alternative online training and standard practice

Journal:	*BMJ Open*
Manuscript ID	bmjopen-2018-022292
Article Type:	Protocol
Date Submitted by the Author:	16-Feb-2018
Complete List of Authors:	Wild, Jennifer; University of Oxford, Experimental Psychology<br>El-Salahi, Shama; University of Oxford, Experimental Psychology<br>Tyson, Gabriella; University of Oxford, Experimental Psychology<br>Lorenz, Hjördis; University of Oxford, Experimental Psychology<br>Pariante, Carmine; King's College London, Psychological Medicine<br>Danese, Andrea; King's College London<br>Tsiachristas, Apostolos; University of Oxford, Health Economics Research Centre<br>Middleton, Benita; University of Surrey, Sleep, Chronobiology and Addiction<br>Blaber, Amanda; University of Brighton, School of Health Sciences<br>Ehlers, Anke; University of Oxford,
Keywords:	Adult psychiatry < PSYCHIATRY, Anxiety disorders < PSYCHIATRY, Depression & mood disorders < PSYCHIATRY

Preventing PTSD, depression, and associated health problems in student paramedics: Protocol for PREVENT-PTSD, a randomised controlled trial of supported online cognitive training for resilience versus alternative online training and standard practice

Jennifer Wild[1,2], Shama El-Salahi[1], Gabriella Tyson[1], Hjördis Lorenz[1], Carmine M. Pariante[3], Andrea Danese[4,5], Apostolos Tsiachristas[6], Benita Middleton[7], Amanda Blaber[8], and Anke Ehlers[1,2]

[1] Oxford Centre for Anxiety Disorders and Trauma, Department of Experimental Psychology, University of Oxford, Oxford, UK
[2] Oxford Health NHS Foundation Trust
[3] Department of Psychological Medicine, Institute of Psychiatry, Psychology and Neuroscience, King's College London, London, UK
[4] Social, Genetic and Developmental Psychiatry, , Institute of Psychiatry, Psychology and Neuroscience, King's College London, London, UK
[5] Department of Child & Adolescent Psychiatry, Institute of Psychiatry, Psychology and Neuroscience, King's College London
[6] Health Economics Research Centre, Nuffield Department of Population Health, University of Oxford, Oxford, UK
[7] Faculty of Health and Medical Sciences, University of Surrey, Guildford, Surrey, UK
[8] School of Health Sciences, University of Brighton, Brighton, UK

Correspondence to: Dr Jennifer Wild; Oxford Centre for Anxiety Disorders and Trauma, The Old Rectory, Paradise Square, Oxford, OX1 1TW, 01865 618614, jennifer.wild@psy.ox.ac.uk

Word count: 5,464

Supplementary Files:

1. **Appendix 1. World Health Organization Trial Registration Data Set.**
2. **Appendix 2. SPIRIT Checklist**

For peer review only

Abstract

Introduction: Emergency workers dedicate their lives to promoting public health and safety, yet suffer higher rates of post-traumatic stress disorder (PTSD) and major depression (MD) compared to the general population. They also suffer an associated increased risk for physical health problems, which may be linked to specific immunological and endocrine markers or changes in relevant markers. Poor physical and mental health is costly to organisations, the NHS and society. Existing interventions aimed at reducing risk of mental ill health in this population are not very successful. More effective preventative interventions are urgently needed. We first conducted a large-scale prospective study of newly recruited student paramedics, identifying two cognitive factors (rumination and resilience appraisals) that predicted episodes of PTSD and MD over a two-year period. We then developed internet-delivered cognitive training for resilience (iCT-R), a supported online intervention, to modify cognitive predictors. This protocol is for a randomised controlled trial to evaluate the efficacy of the resilience intervention.

Methods and analysis: 570 student paramedics will be recruited from participating universities. They will be randomly allocated to iCT-R or to supported online training of an alternative, widely available intervention, or to training-as-usual. Follow-up will occur after the intervention/standard practice period, and at six, 12 and 24 months. We hypothesise that the intervention will lead to reduced rates of subsyndromal and full syndromal PTSD and MD, improved quality of life at the same or even lower cost to the NHS and society, and reduced levels of C-reactive protein (CRP), a marker of inflammation, and the stress hormone cortisol.

For peer review only - http://bmjopen.bmj.com/site/about/guidelines.xhtml

Ethics and dissemination: The Medical Sciences Inter-Divisional Research Ethics Committee at the University of Oxford granted approval, reference: R44116/RE001. The results will be published in a peer-reviewed journal. Access to raw data and participant information will be available only to members of the research team.

Trial registration number: ISRCTN16493616.

Keywords: student paramedics, PTSD, depression, resilience, cortisol, CRP

Strengths and limitations of this study

- The study is a large randomised controlled trial of internet-delivered cognitive training for resilience (iCT-R), which aims to modify cognitive predictors of post-traumatic stress disorder (PTSD) and major depression (MD). Results will indicate whether or not the intervention is associated with lower than expected rates of PTSD and MD over time.
- iCT-R will be evaluated in comparison to an existing online intervention and to standard practice, which will allow determination of intervention-specific changes over time.
- The secondary outcomes of the study will offer opportunity to assess possible neurobiological benefits and cost savings linked to the intervention.
- The two-year follow-up period will allow investigation of the long-term effects of the intervention and its cost-effectiveness.
- Full outcome blinding is not possible.

For peer review only

Introduction

Emergency workers carry a threefold increase, compared to the general population, in risk for major depression (MD) and posttraumatic stress disorder (PTSD), and an associated increased risk of poor physical health.[1] To date, interventions aimed at reducing risk of ill mental health in this population have been unsuccessful. Randomised controlled trials (RCT) found that trauma risk management, a peer support system widely available to the police and ambulance services in England; [2] critical incident stress debriefing widely used by UK fire-services, [3] and the charity Mind's six-session group-based resilience intervention had no effect on resilience or rates of mental ill health [4]. More effective preventative interventions for emergency workers are urgently needed.

Established interventions may have been unsuccessful because they fail to target predictors of mental ill health and are offered to emergency workers after rather than before repeated exposure to the stresses linked to their work. Moreover, cognitive strategies that could help them cope with characteristic stressors are not included as part of the training. For example, our and others' research has demonstrated that exposure to trauma or stressful scenarios through imagery reduces anxiety for police officers and other at risk populations. [5, 6] Development of more effective interventions requires identification of predictors of mental disorders and an understanding of how to modify them.

For peer review only

In a series of experimental and prospective studies, we identified two cognitive factors that are robust predictors of poor mental health in emergency workers: rumination (repetitive negative thinking) and resilience appraisals. Those who reported ruminative thoughts during critical incidents were more likely to experience poor levels of coping. [7] Adaptive appraisals during analogue trauma lead to more successful attempts to regulate emotions and fewer PTSD symptoms. [8] Our large-scale prospective study of newly recruited paramedics investigated predictors of PTSD and MD derived from cognitive theories of PTSD and depression. [1] Rumination at the start of paramedic training uniquely predicted PTSD; low resilience uniquely predicted an episode of MD.

We then developed an intervention to modify peri-traumatic ruminative thinking (i.e, thinking repetitively in an abstract way during trauma). Training to think in a concrete style (e.g., focusing on objective details and the sequence of events) led to significantly fewer intrusive memories and PTSD symptoms than individuals trained in a ruminative style. [9] We also applied one of the core techniques of a successful treatment for PTSD (cognitive therapy for PTSD,[10]), updating the memory of the stressful event with helpful information, as a preventative strategy in analogue trauma and found that it is more helpful in reducing repetitive thinking and PTSD symptoms than control interventions including exposure. [11]

Research has further demonstrated that exposure to trauma or stressful scenarios through imagery reduces anxiety for police officers and other at risk populations,

For peer review only

and that internet-based cognitive treatment that includes attention training as a core component significantly reduces anxiety. [5, 6, 12]

Neurobiological Factors linked to PTSD and MD

Genetic and longitudinal studies suggest that inflammation is a pre-existing vulnerability factor for the development of PTSD in trauma-exposed individuals rather than simply a correlate of subjective distress, disease severity, or maladaptive coping strategies following PTSD onset. [13, 14] For example, brain imaging studies have shown that high inflammation levels may increase threat perception (negative valence). Peripheral administration of lipopolysaccharides (LPS), residues from bacterial cells' components known to elicit a strong systemic inflammatory response, potentiates amygdala activity in response to socially threatening stimuli (fear faces).[15] In turn, greater pre-treatment amygdala reactivity to threat predicts less symptom reduction during CBT.[16] Additionally, inflammation is an important risk factor for depression and cardiovascular disease, which frequently accompany PTSD.[17-19] Our study will investigate the link between inflammation and the development of PTSD and MD in trauma-exposed student paramedics. We will investigate whether or not iCT-R can reduce levels of clinically-relevant inflammation levels, such as C-Reactive Protein (CRP), known to increase risk of psychiatric as well as cardiovascular and metabolic conditions comorbid with PTSD and MD.

Given the wealth of literature supporting a relationship between the stress hormone, cortisol, and PTSD and MD, we will also systematically assess the cortisol awakening response (CAR) and diurnal cycle. The CAR is an endocrine marker,

For peer review only - http://bmjopen.bmj.com/site/about/guidelines.xhtml

defined as the change in cortisol concentration that occurs during the first hour after waking from sleep.[21] A meta-analysis of 62 studies concluded that increases in the CAR were associated with job stress and life stress and linked to greater fatigue, burnout and exhaustion and risk for later health states, such as coronary heart disease.[20] A recent study found that higher CAR predicted future episodes of MD within a 2.5-year period.[21] We anticipate that iCT-R will reduce the CAR and cortisol throughout the day and protect against the development of PTSD and MD.

Study Objectives

The primary aim of the study is to evaluate the efficacy of internet cognitive training for resilience (iCT-R). We hypothesise that iCT-R will lead to fewer cases of PTSD and major depression (including subsyndromal PTSD and MD) and less severe PTSD and MD symptomatology at follow-up compared to an existing online training (Mind-Online) and standard practice.

Secondary Objectives

We hypothesise that iCT-R will lead to greater improvement in secondary outcome measures (resilience, rumination, hormone and immune function, smoking, weight gain, alcohol use, symptoms of anxiety, and sleep problems, psychological distress, wellbeing) than Mind-Online and standard practice. We also expect that iCT-R will be more cost-effective than Mind-Online and standard practice because of lower cost per participant without an episode or with low symptoms of PTSD or MD and lower costs per quality adjusted life years (QALY) gained for participants receiving iCT-R.

For peer review only

Tertiary Objectives

Our tertiary objectives are to determine which psychiatric, personality, trauma and social support factors at baseline (social support, trauma exposure, anxiety, age, gender, education, neuroticism, past and current psychiatric status, immune function) may influence (i.e., moderate) the effect of the interventions on levels of symptoms (PTSD or MD), psychological distress and wellbeing at follow-up. We will also investigate whether or not changes in resilience-related factors (rumination, responses to intrusions, concrete thinking, resilience appraisals, practice of iCT-R/Mind-Online tools) mediate symptom levels of PTSD and MD at one and two-year follow-up with iCT-R and Mind-Online. Finally, we will investigate whether or not concrete thinking, practice of tools and responses to intrusions at 6 months predict diagnoses and levels of PTSD and depression symptoms at one and two-year follow-up.

For peer review only

Methods

This protocol was written in line with the SPIRIT Statement, detailing recommendations for a minimum set of scientific, ethical, and administrative elements that should be addressed in a clinical trial (see Supplementary File).[21]

Design

The proposed study is a single-blind (assessors blinded) randomised controlled trial in which N=570 student paramedics will be randomly allocated to receive internet-delivered cognitive training for resilience (iCT-R), an already available intervention (Mind Online) that has been investigated in previous trials or standard practice. Participants are also invited to give salivary and plasma samples before and after the interventions and at one and two-year follow-up. The trial will take place from October 2017 to January 2021.

Participants

Student paramedics will be recruited from collaborating paramedic training programmes (University of Brighton, Oxford Brookes University, Bournemouth University, University of Hertfordshire, University of Worcester, Kingston University and Anglia Ruskin University). The locations selected constitute rural and city locations to improve generalisability. The researchers will present the study to each year group at collaborating universities to ensure the maximum reach of recruitment. After presenting the study, researchers will collect names and email addresses of interested students and email the registration survey including the participant information sheet (see Supplementary File).

Inclusion and Exclusion Criteria

Students who are aged 18 and above, are training to be paramedics, and are in years 1, 2 or 3 of their paramedic training programme will be eligible for the study. They will be screened for levels of PTSD and MD, and a trained research assistant will contact participants if they score in the clinical range on measures of PTSD or MD, or

For peer review only

report suicidal ideation to evaluate whether they are eligible or need treatment (under JW's supervision). The research assistant will be notified automatically from the screening survey if participants score 10 or above on the total scale or if they score 1 or above on the suicidal ideation item on the Patient Health Questionnaire 9 (PHQ-9)[23] or 33 or above on the Posttraumatic Stress Disorder Checklist for DSM-5 (PCL-5) [24]. Participants will be excluded from the study if their symptoms are interfering with their lives and they would like treatment. The research assistant will offer information on how to access evidence-based treatment for these conditions in local services.

Sample Size Calculation

The risk of student paramedics developing full syndromal PTSD and MD over two years without intervention is 10%, and 25% if subsyndromal PTSD and MD are included [1]. We predict that our intervention will reduce the relative risk by 50%. Setting power at 80%, α=.05 and hypothesizing a reduction of relative risk of 50% gives an odds ratio of 0.429, which requires a total sample size of N=304 to show a risk reduction of 50% between iCT-R and the alternative intervention. Thus, each condition would require N=152. Since we have a third condition (standard practice), the total sample size required would be N=456. Allowing for a 20% rate of attrition, we will require a total sample size of N=570.

Randomisation and blinding

Participants will be randomised on a 1:1:1 ratio as per a computer-generated randomisation schedule stratified by site, gender and baseline PHQ-9 score (≥9

For peer review only - http://bmjopen.bmj.com/site/about/guidelines.xhtml

versus <9) and PCL-5 score (≥33 versus <33). The Oxford Clinical Trials Research Unit is independent to the research team and developed the randomisation programme. The researchers will inform participants of the intervention they are to receive after they have completed baseline assessments. Outcome assessment will be single blind; questionnaires are completed online without any involvement of the researchers, the clinical interview will be conducted by an independent assessor blind to treatment allocation, and all personnel involved in processing and assessing the blood and saliva samples will be blinded from treatment allocation. Due to the nature of the interventions, participants cannot be completely blinded to allocation. However, the inclusion of an already available, alternative intervention aims to mitigate some risk of bias.

Intervention arms

Internet-delivered cognitive training for resilience (iCT-R)

iCT-R aims to modify rumination and appraisals linked to low resilience in a six-session supported online intervention. We include an imagery component, practice of strategies that been shown to prevent stress-related responses from developing,[8, 9] attention training,[12] and weekly top up exercises during follow-up to consolidate training, an approach that is lacking with existing interventions.

Our intervention follows a similar format to the internet-based programmes Clark, Ehlers, Wild and colleagues have developed for social anxiety and PTSD [12, 26]. The core information is delivered in six modules. The modules include whiteboard videos to explain concepts, audio files for practicing concrete thinking, testimonies from

For peer review only

qualified paramedics and video footage of student paramedic call-outs for use in experiential exercises. Following our findings of the protective benefits of concrete thinking [8] and the wealth of work in this area [i.e., 27] participants are regularly reminded to practise concrete thinking.

The modules are:

1. It Matters What you Focus On: Helpful and Unhelpful Attention
2. Get Out of Your Head with Helpful Thinking
3. Habits and Dwelling: How to Change Them
4. Dealing with Unwanted Memories: Then vs Now
5. Transforming Worries & Improving Performance
6. Beating Stress & Trauma: My Blueprint

A trained online coach (research assistant) provides email feedback on students' responses and, through an automated SMS programme, sends regular brief reminders of key points and notifications to practice IF-THEN plans (a technique shown to help individuals respond to warning signs of stress and dwelling).

Mind Online

The alternative intervention is a series of six modules available online covering information and advice about stress, sleep problems, anger, depression, PTSD and mindfulness. Participants will receive the same frequency, type and duration of remote support as in iCT-R.

Standard Practice

For peer review only

The third condition is training as usual. Participants will have access to the usual support offered through their university but they will not receive any online modules or remote support. They will be offered the iCT-R at the end of follow-up, when the study is completed.

Primary Outcome Measures

Levels of PTSD and MD

An independent assessor will administer the PTSD and MD modules of the Structured Clinical Interview for DSM-5 (SCID-5) to assess clinical and subsyndromal cases of PTSD and MD [28]. The SCID-5 is an interview schedule for determining DSM-5 psychiatric diagnoses. PTSD and MD symptomatology will also be assessed with continuous measures: the PCL-5 and the PHQ-9 [23, 24]. The PCL-5 is a self-report measure consisting of 20 questions that parallel the diagnostic criteria for PTSD set out in the Diagnostic and Statistical Manual of Mental Disorders, 5th edition [29]. Items are scored on a 5-point Likert scale (0 = "Not at all" to 4 = "Extremely"). The PHQ-9 is a well-validated 9-item self-report measure that assesses symptoms of depression. Items are scored on a 4-point Likert scale (0 = "Not at all" to 3 = "Nearly every day").

Secondary Outcomes

Psychological outcomes

Two measures of resilience will be administered: the Wagnild Resilience Scale and the Connor-Davidson Resilience Questionnaire (CD-RISC). [30, 31] The Wagnild Resilience Scale is a 25-item scale that measures resilience by rating responses to

For peer review only - http://bmjopen.bmj.com/site/about/guidelines.xhtml

statements on a 7-point Likert scale (0 = “Strongly disagree” to 7 = “Strongly agree”). The CD-RISC is a commonly used self-report measure of resilience with 25 items scored on a 5-point Likert scale (0 = “Not true at all” to 4 = “True nearly all the time”). Both scales are well-validated measures with excellent psychometric properties. Rumination will be assessed with the brooding subscale of the Ruminative Responses Scale (RRS), a reliable and valid measure of the frequency of engaging in dwelling [32]. Rumination in response to unwanted memories will be assessed with the dwelling subscale of the Responses to Intrusions Questionnaire (RIQ), a reliable and valid measure of maladaptive responses to intrusive memories [33]. Anxiety will be measured with the Generalized Anxiety Disorder scale 7 (GAD-7), a 7-item scale with items scored on a 4-point Likert scale (0 = “Not at all” to 3 = “Nearly every day”) [34]. Psychological distress will be measured with the reliable and valid 12-item General Health Questionnaire 12 (GHQ-12) [35]. Wellbeing will be assessed using the Warwick Edinburgh Mental Wellbeing Scale (WEMWBS) [36]. The WEMWBS has 14 items and is scored on a 5-point Likert scale (1= “None of the time” to 5 = “All of the time”).

Hormone and immune function

Salivary cortisol will be assessed by RIA assay analysis. A sample of saliva will be collected upon awakening, 15, 30 and 60 minutes after awakening, and at 12 noon and 8pm. Baseline high-sensitive CRP plasma levels will be measured using an ILab 600 spectrophotometric method in serum samples.

Health outcomes

For peer review only

Smoking and alcohol use will be measured with unpublished questionnaires. Participants will be asked to indicate whether or not they smoke, how many cigarettes they smoke a day, and whether this has increased, decreased, or stayed the same in the last year. They will also be asked how many units of alcohol they have had in the last week, whether this is an average amount for them, and if not, how many units they usually drink per week. Weight gain will be measured by increases in BMI. Participants will be asked to provide their weight and height. The researchers will take weighing scales and a tape measure to study visits to weigh and measure participants. Sleep problems will be assessed by the Insomnia Severity Index (ISI), which is a reliable and valid brief self-report instrument of sleep quality and sleep difficulties.[37] In line with NICE guidelines, health related quality of life will be measured by the five-levels version of the EuroQol 5 Dimensions questionnaire (EQ-5D-5L).[38] The EQ-5D-5L is a validated and widely used generic measurement of health related quality of life based on five dimensions: mobility, self-care, usual activities, pain/discomfort, and anxiety/depression. Although, we do not expect that all five dimensions will be affected by the intervention, we need to collect data on all of them dimensions in order to determine quality adjusted life years (QALYs).

Costs

The economic evaluation will be conducted by taking the NHS perspective (including the costs of using mental health services) and the broader societal perspective (including additionally the costs of productivity loss due to illness). Mental health resource utilisation will be measured with an adapted version of the Clinical Service

For peer review only

Receipt Inventory (CSRI) and valued by using published unit costs (e.g. NHS Reference Costs and Unit Costs of Health and Social Care).[39]. Productivity loss will be measured with Short Form-Health and Labor Questionnaire (SF-HLQ), a validated questionnaire part of the Trimbos/iMTA questionnaire for Costs associated with Psychiatric Illness (TiC-P).[37, 39]

Process Analyses

To assess potential moderators of outcomes, we will measure psychiatric, personality, trauma and social support factors at baseline (social support, trauma exposure, anxiety, age, gender, education, neuroticism, past and current psychiatric status, immune function). The neuroticism subscale (12 items) of the Eysenck Personality Questionnaire (EPQ) has excellent psychometric properties and is a measure of emotionality [43]. We will use an adapted version of a brief measure of social support (SS), to assess perceived support from and closeness to friends, family and work colleagues, as well as use of social support [44]. Trauma exposure will be measured using a 19-item unpublished trauma questionnaire relevant to emergency workers, which includes items from the Clinician Administered PTSD Scale (CAPS) [45]. We will also collect demographic information (age, gender, and level of education), information on the duration, frequency and distress linked to intrusions, and questions about concrete and abstract thinking based on an existing assessment tool [46]. Participants will be asked to think about a problem they are having and write questions that may go through their minds in relation to the problem. They will then be presented with four problem scenarios and asked to select from a list of the likely thoughts they would have if faced with the problem. The list consists of a range

For peer review only - http://bmjopen.bmj.com/site/about/guidelines.xhtml

of concrete and abstract thoughts. We will investigate whether or not changes in resilience-related factors (rumination, responses to intrusions, concrete thinking, resilience appraisals, practice of iCT-R/Mind-Online tools) mediate symptom levels of PTSD and MD at one and two-year follow-up with iCT-R and Mind-Online. We will also investigate whether or not concrete thinking, practice of tools and responses to intrusions at 6 months predict diagnoses and levels of PTSD and depression symptoms at one and two-year follow-up. See Table 1 for the full list of outcomes, measures and assessment time points.

For peer review only

Table 1. Outcomes and measures

	Domain	Measures	Time point*
Primary Outcomes	PTSD	Structured Clinical Interview for DSM-5 Disorders (SCID-5).[28]<br>Post-traumatic Stress Disorder Checklist (PCL-5).[23]	0 1 3 4<br>0 1 2 3 4
	MD	Structured Clinical Interview for DSM-V Disorders (SCID-5).[28].<br>Patient Health Questionnaire 9 (PHQ-9).[23]	0 1 3 4<br>0 1 2 3 4
Secondary Outcomes	Resilience	Connor-Davidson Resilience Questionnaire (CD-RISC).[30]<br>Wagnild Resilience Scale.[31]	0 1 3 4<br>0 1 3 4
	Rumination	Ruminative Responses Scale (RRS) brooding subscale.[32]<br>Responses to Intrusions Questionnaire (RIQ) dwelling subscale.[33]	0 1 3 4
	Anxiety	Generalized Anxiety Disorder 7-item scale (GAD-7).[33]	0 1 3 4
	Smoking and Alcohol use	Smoking Behaviour Questionnaire (unpublished).<br>Alcohol Use Questionnaire (unpublished).	0 1 3 4
	Weight and Height	Unpublished questionnaire recording participants' height and weight.	0 1 3 4
	Psychological distress	General Health Questionnaire (GHQ-12).[35]	0 1 3 4
	Wellbeing	Warwick Edinburgh Mental Wellbeing Scale (WEMWBS).[36]	0 1 3 4
	Hormone function	Level of cortisol in response to awakening and throughout the day.	0 1 3 4
	Immune function	Level of C-reactive protein.	0 1 3 4
	Sleep problems	Insomnia Severity Index (ISI)[37]	0 1 3 4
	Health economics	EuroQol (EQ-5D-5L).[38]<br>Trimbos/iMTA Questionnaire for Costs Associated with Psychiatric Illness (TiC-P).[40]<br>Client Service Receipt Inventory.[39, 41]<br>Health and Labor Questionnaire [42]	0 1 3 4<br>0 1 3 4<br><br>0 1 3 4<br>0 1 3 4
Tertiary Outcomes	Neuroticism	Eysenck Personality Questionnaire (EPQ) neuroticism subscale.[43]	0 1 3 4
	Social support	Social Support scale (SS) adapted from a brief measure of social support.[44]	0 1 3 4
	Demographics	General information questionnaire (unpublished).	0 1 3 4
	Trauma exposure	Trauma Screener (unpublished).	0 1 2 3 4
	Concrete thinking	Concrete thinking questionnaire, adapted from a previous concrete thinking assessment.[46]	0 1 2 3 4
	Intrusions	Duration, frequency and distress linked to intrusions questionnaire (unpublished)	0 1 2 3 4

*Timepoint: 0 = baseline, 1 = post-intervention, 2 = 6 month follow-up, 3 = 12 month follow-up, 4 = 24 month follow-up

Procedure

Researchers will present the study at collaborating universities and invite student paramedics to take part. Interested participants will be given a weblink to the study via our software platform, Qualtrics, where they can read and print a PDF copy of

For peer review only - http://bmjopen.bmj.com/site/about/guidelines.xhtml

the Participant Information Sheet and discuss questions with the research assistant over the telephone. If they decide to take part, they will be emailed a link where they can login, re-read the Participant Information Sheet and complete a consent form (see Supplementary File for the Participant Information Sheet and Consent Form). Written consent will be requested from participants before blood and saliva samples are taken. It will be made clear that participation is entirely voluntary and that volunteers may withdraw from the study at any point without incurring any negative consequences.

Participants will be recruited over a 12 month period. They will complete online two questionnaires (PCL-5 and PHQ-9) to assess eligibility. If participants score ≥9 on the PHQ-9, ≥38 on the PCL-5 or ≥1 on the PHQ-9 suicidal ideation item, a researcher will phone the participant to determine symptom severity and whether treatment is necessary, and conduct a risk assessment if necessary. If treatment is needed, participants will be signposted to their GP and local psychological services. If participants are eligible and consent to participate in the study, they will be assigned a participant ID number to ensure anonymity is maintained. They will complete a set of questionnaires online, take part in a clinical interview conducted by an independent assessor, and provide blood and saliva samples. The clinical interviews will be recorded on SanDisk MP3 recorders to ensure that the questions asked are standardised across all assessment interviews. Qualified phlebotomists will visit students at their universities to collect blood samples. During this study visit, participants will be provided with equipment to collect their saliva at home upon awakening and through the day (6 samples in total). Saliva and blood samples will be

transported to the University of Surrey for assay analysis. The blood samples will be centrifuged immediately and only serum will be kept for analysis.

Once baseline assessments are completed, participants will be randomly allocated to iCT-R, Mind-Online or standard practice. When the interventions or standard practice are completed, participants will complete the full battery of assessments again; they will complete the questionnaires online, take part in a clinical interview, and provide blood and saliva samples. Six months later, they will complete online a shorter set of questionnaires. At 12 and 24-month follow-up, they will complete the full battery of assessments again. See Figure 1 for a timeline of the study including the enrolment process, randomisation, interventions and assessments. To discourage discontinuation, participations will be compensated with £30 on completion of questionnaires at follow up time points. The reasons for non-adherence to the intervention or dropping out of the study will be recorded. An independent rater will assess treatment fidelity. The content of a random sample of email communications will be scored for reference to content relevant to each training programme.

For peer review only

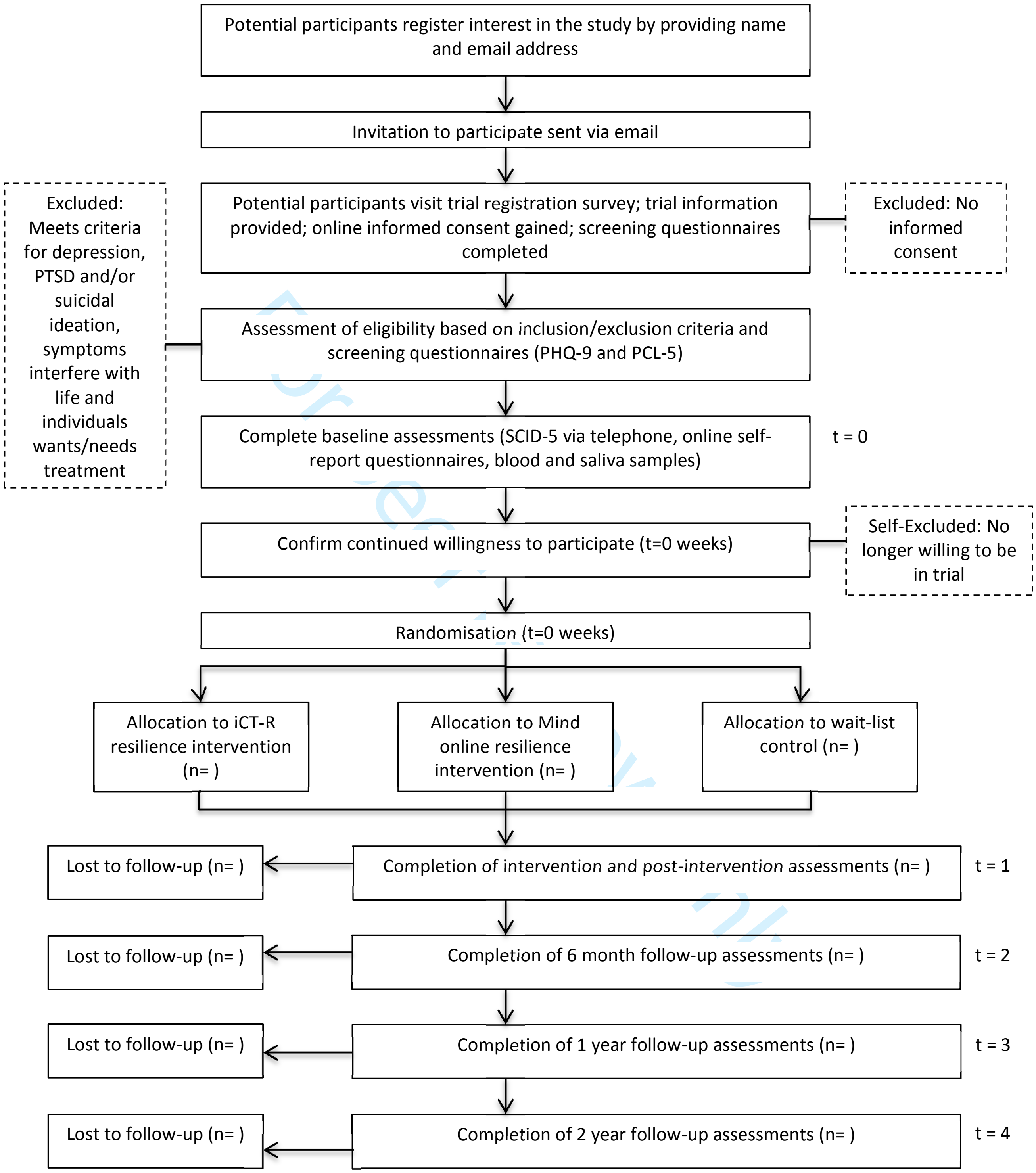

Fig 1. Study timeline.

For peer review only - http://bmjopen.bmj.com/site/about/guidelines.xhtml

Data Management

Registration and assessment data will be captured online via Qualtrics software. Participants will be assigned a unique code to be used for all data files and audio tapes. Access to the system will be restricted to named study personnel and via password protection. The University of Oxford's IT services have arranged for the files to be encrypted and backed up on a weekly basis using a Tivoli Storage Manager (TSM). The data will be copied to three separate tapes; one copy will reside in the Tape Robot in the IT Services Machine room and the other two are held in locked fireproof safes, one onsite at IT services and one offsite in locked premises. The data on the tapes are inaccessible without the TSM database. The data on the offsite tapes are encrypted. Papers from clinical interviews will be kept in locked cabinets at the University of Oxford. The audiotapes from clinical interviews will be backed up online with password-protection and access restricted to study personnel. The blood samples will be centrifuged as soon as the laboratory at the University of Surrey receives them on the day of collection. The cellular component will be discarded and the serum will be stored at -80 °C. Saliva samples will be analysed at the University of Surrey by RIA assay analysis to detect levels of cortisol.

Statistical Analyses

In line with the BMJ and Consort guidelines, data analysis will be intent-to-treat. All participants who have been randomised will be included in analyses, including those who drop out. We will compare rates of PTSD and MD in each condition using Chi square analysis. Continuous measures will be analysed using hierarchical linear modelling. This analysis models random slopes and intercepts for participants, and

For peer review only - http://bmjopen.bmj.com/site/about/guidelines.xhtml

tests the fixed effects of repeated assessments over time (level 1) and treatment condition (level 2) using data from all participants. It takes into account that participants are nested within site (level 3). Variables will be centred for the analysis.

Additional analyses will investigate potential interactions between treatment effects and candidate moderators such as social support, exposure to critical incidents, and baseline CRP. Mediation analyses will be conducted to assess whether or not changes in resilience-related factors (rumination, responses to intrusions, concrete thinking, resilience appraisals, practice of iCT-R/Mind-Online tools) mediate symptom levels of PTSD and MD at one and two-year follow-up with iCT-R and Mind-Online.

A trial-based economic evaluation will be conducted to investigate the cost-effectiveness of the intervention in terms of cost per QALY gained. Uncertainty in the results will be addressed in sensitivity analyses and displayed in cost-effectiveness planes and cost-effectiveness acceptability curves.

Adverse Events

We do not anticipate any adverse events. However, it is possible that a participant may evidence risk at one of the assessment points (pre-intervention, post-intervention, or 12 and 24-month follow-up). If this is the case, risk will be assessed over the telephone and the individual will be signposted to the appropriate service.

Should a serious adverse event (SAE) occur where, in the opinion of the Principal

For peer review only - http://bmjopen.bmj.com/site/about/guidelines.xhtml

Investigator, the event was 'related' (resulted from administration of any of the research procedures) and 'unexpected' in relation to those procedures, it will be reported to the Research Ethics Committee. Reports of related and unexpected SAEs will be submitted within 15 working days of the Principal Investigator becoming aware of the event, using the Health Research Authority safety report form for a non-Clinical Trial of an Investigation of a Medicinal Product (non-CTIMP).

The University of Oxford has a specialist insurance policy in place, which would operate in the event of any participant suffering harm as a result of their involvement in the research (Newline Underwriting Management Ltd, at Lloyd's of London).

ETHICS AND DISSEMINATION

Ethical approval of the research protocol was gained from The Medical Sciences Inter-Divisional Research Ethics Committee at the University of Oxford, 17/08/2017, ref: R44116/RE001. This is protocol version 1. Any substantive amendments to the protocol will be conducted by the Principal Investigator and reviewed by the Research Ethics Committee.

The research results will be submitted for publication in a peer-reviewed journal and presented at relevant conferences. Direct access to data will be granted to

For peer review only

authorised representatives from the host institution and the regulatory authorities to permit trial-related monitoring, audits and inspections.

Committees. We have established a Trial Oversight Committee (TOC). Our independent chairman is Dr Susan Dutton, the Senior Medical Statistician and Oxford Clinical Trials Research Unit Lead Statistician. The Principal Investigator is also a member of the TOC and we have one lay qualified paramedic member (Graham Harris). The TOC will meet before the start of the trial and three more times before the end of the trial.

Acknowledgements. We are grateful to Dr Esther Beierl for conducting the independent statistical analyses for this study, and to Dr Susan Dutton for chairing the Trial Oversight Committee.

Contributors. JW is the Principal Investigator and will oversee the study and arrange and lead meetings with a User Advisory Group and the Trial Oversight Committee. JW conceived and co-designed the study with AE. JW developed the intervention with AE, Edward Watkins, and GT. JW completed the ethics application, and will liaise with collaborators to support recruitment, manage and supervise the research team and write the primary paper for publication. AB will facilitate recruitment and contact with collaborating centres. GT will offer online support to participants receiving the online interventions and help to schedule assessments. SE will be the independent assessor at baseline and follow-up. JW, GT, SE and HL are responsible

For peer review only

for data collection. BM will analyse the biological data. CP, AD and AT will guide the analyses of the biological and health economics data.

Funding. This work is funded by an MQ: Transforming Mental Health grant (number CQR01260) and supported by the NIHR Oxford Health Biomedical Research Centre. MQ had no role in the design of this study and will not have any role during its execution, analyses, interpretation of the data, or decision to submit results. Anke Ehlers is funded by a Wellcome Trust Principal Research Fellowship (grant 200796). CMP is supported by the NIHR Biomedical Research Centre at the South London and Maudsley NHS Trust and King's College London, London, UK. The views expressed are those of the authors and not necessarily those of the NHS, the NIHR or the Department of Health.

Competing interests. Dr Jennifer Wild, Professor Anke Ehlers and their team have developed iCT-R. They do not receive any income from this work.

For peer review only

References

1 Wild J, Smith KV, Thompson E, et al. A prospective study of pre-trauma risk factors or posttraumatic stress disorder and depression. *Psychol Med* 2016;46(1):2571-82.

2 Greenberg N, Langston V, Everitt, B., Iversen, A., Fear, N.T., Jones, N. & Wessely, S. (2010). A cluster randomised controlled trial to determine the efficacy of trauma risk management (TRiM) in a military population. *J Trauma Stress, 23,* 430-36.

3 van Emmerik AA, Kamphuis JH, Hulsbosch AM, Emmelkamp PM (2002). Single session debriefing after psychological trauma: a meta-analysis. *Lancet* 2002;360:766-71.

4 Wild J, El-Salahi S, Degli Esposti M, & Ehlers, A. (Under review). A randomised controlled trial of an existing group-based resilience intervention for emergency workers.

5 Arnetz BB, Arble E, Backman L, Lynch A, & Lublin A. Assessment of a prevention program for work-related stress among urban police officers. *Int Arch Occup Environ Health* 2013;86:79-88.

6 Wild J, Hackmann A, Clark DM. Rescripting early memories linked to negative images in social phobia: A pilot study. *Behav Ther* 2008;39:47-56.

7 Shepherd L, Wild J. Cognitive appraisals, objectivity, and coping in ambulance workers: a pilot study. *Emerg Med J* 2013;31:41-4.

8 Shepherd L, Wild J. Emotion regulation, physiological arousal and PTSD symptoms in trauma-exposed individuals. *J Behav Ther Exp Psychiatry* 2014;45:360-7.

9 White R, Wild J. “Why” or “How”: The effect of concrete versus abstract processing on intrusive memories following analogue trauma. *Behav Ther* 2016:47(3):404-415.

10 Ehlers A., Hackmann A., Grey N, Wild J, et al. A randomized controlled trial of 7-day intensive and standard weekly cognitive therapy for PTSD and emotion-focused supportive therapy. *Am J Psychiatry,* 2014:171, 294-304.

11 Pile V, Barnhofer T, Wild J. Updating versus exposure to prevent consolidation of conditioned fear. *PLoS One* 2015;10:1-21.

12 Stott R, Wild J, Grey N et al. Internet-delivered cognitive therapy for social anxiety disorder: a development series. *Behav Cogn Psychother* 2014;41:383-97.

13 Michopoulos V, Rothbaum AO, Jovanovic T, et al. Association of CRP genetic variation and CRP level with elevated PTSD symptoms and physiological responses in a civilian population with high levels of trauma. *Am J Psychiatry,* 2015;172:353-362.

For peer review only

14 Eraly SA, Nievergelt CM, Maihofer AX, et al. Assessment of plasma C-reactive protein as a biomarker of posttraumatic stress disorder risk. *JAMA Psychiatry* 2014;71:423-431.

15 Inagaki TK, Keely A, Muscatell MA, et al. Inflammation Selectively Enhances Amygdala Activity to Socially Threatening Images. *Neuroimage 2012;*15:3222-6.

16 Bryant RA, Felmingham K, Kemp A, et al. Amygdala and ventral anterior cingulate activation predicts treatment response to cognitive behaviour therapy for post-traumatic stress disorder. *Psychol Med* 2008;*38:*555-61.

17 Miller AH, Raison CL. The role of inflammation in depression: from evolutionary imperative to modern treatment target. *Nat Rev in Immunol* 2016;16:22-34.

18 Ridker PM, Cushman M, Stampfer MJ, et al. Inflammation, Aspirin, and the Risk of Cardiovascular Disease in Apparently Healthy Men. *N Engl J Med* 1997;336:973-9.

19 Sumner JA, Kubzansky LD, Elkind MS, et al. Trauma Exposure and Posttraumatic Stress Disorder Symptoms Predict Onset of Cardiovascular Events in Women. *Circulation* 2015;8:251-9.

20 Chida Y, Steptoe A. Cortisol awakening response and psychosocial factors: a systematic review and meta-analysis. *Biol Psychiatry* 2009;80:265-78.

21 Vrshek-Schallhorn S, Doane LD, Mineka S, et al. The cortisol awakening response predicts major depression: predictive stability over a 4-year follow-up and effect of depression history. *Psychol Med* 2013;43:483-93.

22 Chan AW, Tetzlaff JM, Altman DG, et al. SPIRIT 2013 Statement: Defining standard protocol items for clinical trials. *Ann Intern Med* 2013;158:200-7.

23 Kroenke K, Spitzer R, Williams JB. The PHQ-9. Validity of a Brief Depression Severity Measure. *J Gen Intern Med* 2001;16:603-13.

24 Weathers FW, Litz BT, Keane TM, et al. *The PTSD Checklist for DSM-5 (PCL-5)* 2013. Scale available from the National Center for PTSD at www.ptsd.va.gov.

25 Olff M, de Vries GJ, Guzelcan Y, et al. Changes in cortisol and DHEA plasma levels after psychotherapy for PTSD. *Psychoneuroendocrinology* 2007;32:619-26.

26 Wild J, Warnock-Parkes E, Grey N, et al. Internet-delivered cognitive therapy for PTSD: a development pilot series. *Eur J Psychotraumatol* 2016;7:10.3402/ejpt.v7.31019. doi:10.3402/ejpt.v7.31019.

27 Watkins E, Taylor RS, Byngs R, et al. Guided self-help concreteness training as an intervention for major depression in primary care: a Phase II randomized controlled trial. *Psychol Med* 2011;*42:*1359-71.

For peer review only

28 First MB, Williams JBW, Karg RS, et al. Structured Clinical Interview for DSM-5, Clinician Version (SCID-5-CV). Arlington, VA: American Psychiatric Association, 2016.

29 American Psychiatric Association. Diagnostic and statistical manual of mental disorders (5th ed.). Arlington, VA: American Psychiatric Publishing, 2013.

30 Connor KM, Davidson JRT. Development of a new resilience scale: the Connor-Davidson Resilience Scale (CD-RISC). *Depress Anxiety* 2003;18:76-82.

31 Wagnild GM, Young HM. Development and psychometric evaluation of the Resilience Scale. *J Nurs Meas* 1993;1(2):165-78.

32 Treynor W, Gonzalez R, Nolen-Hoeksema S. Rumination reconsidered: a psychometric analysis. *Cognit Ther Res* 2003;27:247-59.

33 Clohessy S, Ehlers A. PTSD symptoms, response to intrusive memories, and coping in ambulance service workers. *Br J Clin Psychol* 1999;38:251-65.

34 Spitzer RI, Kroenke K, Williams JW et al. A brief measure for assessing generalized anxiety disorder: The GAD-7. *Arch Intern Med* 2006;166(10):1092-7.

35 Goldberg D, Williams P. A user's guide to the General Health Questionnaire. Windsor, UK: NFER-Nelson 1988.

36 Tennant R, Hiller L, Fishwick R, et al. The Warwick-Edinburgh Mental Well-being Scale (WEMWBS): development and UK validation. *Health Qual Life Outcomes* 2007;5:63.

37 Morin CM, Belleville G, Bélinger L, et al. The Insomnia Severity Index: psychometric indicators to detect insomnia cases and evaluate treatment response. *Sleep* 2011;34(5):601-8.

38 EuroQol: the current state of play. *Health Policy* 1996;37:53-72.

39 Chisholm D, Knapp MR, Knudsen HC, et al. Client Socio-Demographic and Service Receipt Inventory--European Version: development of an instrument for international research. EPSILON Study 5. European Psychiatric Services: Inputs Linked to Outcome Domains and Needs. *Br J Psychiatry Suppl*. 2000(39):s28-33.

40 Hakkaart-van Roijen L, Van Straten A, Donker M, et al. Manual Trimbos/iMTA questionnaire for Costs associated with Psychiatric Illness (TiC-P). Rotterdam: Institute for Medical Technology Assessment, Erasmus MC Rotterdam 2002.

41 Chisholm D, Knapp MR, Knudsen HC, et al. Client Socio-Demographic and Service Receipt Inventory--European Version: development of an instrument for

For peer review only

international research. EPSILON Study 5. European Psychiatric Services: Inputs Linked to Outcome Domains and Needs. *Br J Psychiatry Suppl*. 2000(39):s28-33.

42 Roijen VL, Essink-Bot M, Koopmanschap M, et al. Labor and health status in economic evaluation of health care. The Health and Labor Questionnaire. *Int J Technol Assess Health Care* 1996;12(3):405-415.

43 Eysenck HJ, Eysenck SBG. Manual of the Eysenck Personality Questionnaire. London: Hodder & Stoughton 1975.

44 Sarason IG, Sarason BR, Shearin EN, et al. A brief measure of social support: Practical and Theoretical Implications. *J Soc Pers Relat* 1987:4:497-510.

45 Weathers FW, Blake DD, Schnurr PP, et al. *The Clinician-Administered PTSD Scale for DSM-5 (CAPS-5)*, 2013. Interview available from the National Center for PTSD at www.ptsd.va.gov.

46 Stöber J, Borkovec TD. Reduced concreteness of worry in generalized anxiety disorder: Findings from a therapy study. *Cognit Ther Res* 2002;26:89–96.

For peer review only

APPENDICES

Appendix 1. World Health Organization Trial Registration Data Set.
Appendix 2. SPIRIT Checklist.

For peer review only

Appendix 1. World Health Organization Trial Registration Data Set.

DATA CATEGORY	INFORMATION
Primary registry and trial identifying number	Registry: ISRCTN. Identifying number: ISRCTN16493616.
Date of registration in primary registry	9 October, 2017
Secondary identifying numbers	Protocol/serial number: PREVENT-PTSD/Protocol V1.
Source(s) of monetary or material support	MQ: Transforming Mental Health
Primary sponsor	MQ: Transforming Mental Health
Secondary sponsor(s)	University of Oxford
Contact for public queries	*JW* +44(0)1865 618612, jennifer.wild@psy.ox.ac.uk
Contact for scientific queries	*JW* +44(0)1865 618612, jennifer.wild@psy.ox.ac.uk
Public title	PREVENT-PTSD
Scientific title	Preventing PTSD, depression, and associated health problems in student paramedics: Protocol for a parallel-group randomised controlled trial of a supported online resilience intervention versus a placebo
Countries of recruitment	England
Health condition(s) or problem(s) studied	Post-traumatic stress disorder (PTSD), major depression (MD), resilience, adverse health behaviours (sleep problems, weight gain and smoking), cost savings in NHS and society, cortisol, inflammation levels
Intervention(s)	Active comparator: Resilience intervention focusing on modifying rumination and cognitive appraisals<br>Placebo comparator: Online information and advice about stress, sleep problems, anger, depression PTSD and mindfulness<br>Wait-list comparator: Resilience intervention delivered after the two-year study
Key inclusion and exclusion criteria	Ages eligible for study: ≥18 years<br>Sexes eligible for study: both<br>Accepts healthy volunteers: yes<br>Inclusion criteria: student paramedics (≥ 18 years) who do not currently have depression or PTSD needing treatment<br>Exclusion criteria: student paramedics who meet criteria for depression or PTSD and whose lives are significantly impacted by their symptoms, thereby needing treatment
Study type	Interventional<br>Allocation: randomised<br>Intervention model: parallel assignment<br>Masking: assessors blinded to all assessments<br>Primary purpose: prevention<br>Phase I
Date of first enrolment	October 2017
Target sample size	570
Recruitment status	Recruiting and delivering first wave of interventions

For peer review only

Primary outcome(s)	Diagnoses of PTSD and MD assessed by a blind researcher using the Structured Clinical Interview for DSM-5
Key secondary outcomes	Mental Health: resilience, rumination, symptoms of anxiety, and sleep problems, psychological distress, wellbeing<br><br>Immune and Endocrine Function: cortisol awakening response and levels of C-reactive protein<br><br>Physical health behaviours: smoking, weight gain, alcohol use.<br><br>Health Economics: utilisation of health services, episodes of PTSD and MD, absenteeism and presenteeism

For peer review only

Appendix 2. SPIRIT Checklist.

SPIRIT

STANDARD PROTOCOL ITEMS: RECOMMENDATIONS FOR INTERVENTIONAL TRIALS

SPIRIT 2013 Checklist: Recommended items to address in a clinical trial protocol and related documents*

Section/item	Item No	Description
**Administrative information**		
Title	1	Descriptive title identifying the study design, population, interventions, and, if applicable, trial acronym
Trial registration	2a	Trial identifier and registry name. If not yet registered, name of intended registry
	2b	All items from the World Health Organization Trial Registration Data Set
Protocol version	3	Date and version identifier
Funding	4	Sources and types of financial, material, and other support
Roles and responsibilities	5a	Names, affiliations, and roles of protocol contributors
	5b	Name and contact information for the trial sponsor
	5c	Role of study sponsor and funders, if any, in study design; collection, management, analysis, and interpretation of data; writing of the report; and the decision to submit the report for publication, including whether they will have ultimate authority over any of these activities
	5d	Composition, roles, and responsibilities of the coordinating centre, steering committee, endpoint adjudication committee, data management team, and other individuals or groups overseeing the trial, if applicable (see Item 21a for data monitoring committee)
**Introduction**		
Background and rationale	6a	Description of research question and justification for undertaking the trial, including summary of relevant studies (published and unpublished) examining benefits and harms for each intervention
	6b	Explanation for choice of comparators
Objectives	7	Specific objectives or hypotheses

For peer review only

Trial design	8	Description of trial design including type of trial (eg, parallel group, crossover, factorial, single group), allocation ratio, and framework (eg, superiority, equivalence, noninferiority, exploratory)
**Methods: Participants, interventions, and outcomes**		
Study setting	9	Description of study settings (eg, community clinic, academic hospital) and list of countries where data will be collected. Reference to where list of study sites can be obtained
Eligibility criteria	10	Inclusion and exclusion criteria for participants. If applicable, eligibility criteria for study centres and individuals who will perform the interventions (eg, surgeons, psychotherapists)
Interventions	11a	Interventions for each group with sufficient detail to allow replication, including how and when they will be administered
	11b	Criteria for discontinuing or modifying allocated interventions for a given trial participant (eg, drug dose change in response to harms, participant request, or improving/worsening disease)
	11c	Strategies to improve adherence to intervention protocols, and any procedures for monitoring adherence (eg, drug tablet return, laboratory tests)
	11d	Relevant concomitant care and interventions that are permitted or prohibited during the trial
Outcomes	12	Primary, secondary, and other outcomes, including the specific measurement variable (eg, systolic blood pressure), analysis metric (eg, change from baseline, final value, time to event), method of aggregation (eg, median, proportion), and time point for each outcome. Explanation of the clinical relevance of chosen efficacy and harm outcomes is strongly recommended
Participant timeline	13	Time schedule of enrolment, interventions (including any run-ins and washouts), assessments, and visits for participants. A schematic diagram is highly recommended (see Figure)
Sample size	14	Estimated number of participants needed to achieve study objectives and how it was determined, including clinical and statistical assumptions supporting any sample size calculations
Recruitment	15	Strategies for achieving adequate participant enrolment to reach target sample size
**Methods: Assignment of interventions (for controlled trials)**		
Allocation:		
Sequence generation	16a	Method of generating the allocation sequence (eg, computer-generated random numbers), and list of any factors for stratification. To reduce predictability of a random sequence, details of any planned restriction (eg, blocking) should be provided in a separate document that is unavailable to those who enrol participants or assign interventions

Allocation concealment mechanism	16b	Mechanism of implementing the allocation sequence (eg, central telephone; sequentially numbered, opaque, sealed envelopes), describing any steps to conceal the sequence until interventions are assigned
Implementation	16c	Who will generate the allocation sequence, who will enrol participants, and who will assign participants to interventions
Blinding (masking)	17a	Who will be blinded after assignment to interventions (eg, trial participants, care providers, outcome assessors, data analysts), and how
	17b	If blinded, circumstances under which unblinding is permissible, and procedure for revealing a participant's allocated intervention during the trial
**Methods: Data collection, management, and analysis**		
Data collection methods	18a	Plans for assessment and collection of outcome, baseline, and other trial data, including any related processes to promote data quality (eg, duplicate measurements, training of assessors) and a description of study instruments (eg, questionnaires, laboratory tests) along with their reliability and validity, if known. Reference to where data collection forms can be found, if not in the protocol
	18b	Plans to promote participant retention and complete follow-up, including list of any outcome data to be collected for participants who discontinue or deviate from intervention protocols
Data management	19	Plans for data entry, coding, security, and storage, including any related processes to promote data quality (eg, double data entry; range checks for data values). Reference to where details of data management procedures can be found, if not in the protocol
Statistical methods	20a	Statistical methods for analysing primary and secondary outcomes. Reference to where other details of the statistical analysis plan can be found, if not in the protocol
	20b	Methods for any additional analyses (eg, subgroup and adjusted analyses)
	20c	Definition of analysis population relating to protocol non-adherence (eg, as randomised analysis), and any statistical methods to handle missing data (eg, multiple imputation)
**Methods: Monitoring**		
Data monitoring	21a	Composition of data monitoring committee (DMC); summary of its role and reporting structure; statement of whether it is independent from the sponsor and competing interests; and reference to where further details about its charter can be found, if not in the protocol. Alternatively, an explanation of why a DMC is not needed

	21b	Description of any interim analyses and stopping guidelines, including who will have access to these interim results and make the final decision to terminate the trial
Harms	22	Plans for collecting, assessing, reporting, and managing solicited and spontaneously reported adverse events and other unintended effects of trial interventions or trial conduct
Auditing	23	Frequency and procedures for auditing trial conduct, if any, and whether the process will be independent from investigators and the sponsor
**Ethics and dissemination**		
Research ethics approval	24	Plans for seeking research ethics committee/institutional review board (REC/IRB) approval
Protocol amendments	25	Plans for communicating important protocol modifications (eg, changes to eligibility criteria, outcomes, analyses) to relevant parties (eg, investigators, REC/IRBs, trial participants, trial registries, journals, regulators)
Consent or assent	26a	Who will obtain informed consent or assent from potential trial participants or authorised surrogates, and how (see Item 32)
	26b	Additional consent provisions for collection and use of participant data and biological specimens in ancillary studies, if applicable
Confidentiality	27	How personal information about potential and enrolled participants will be collected, shared, and maintained in order to protect confidentiality before, during, and after the trial
Declaration of interests	28	Financial and other competing interests for principal investigators for the overall trial and each study site
Access to data	29	Statement of who will have access to the final trial dataset, and disclosure of contractual agreements that limit such access for investigators
Ancillary and post-trial care	30	Provisions, if any, for ancillary and post-trial care, and for compensation to those who suffer harm from trial participation
Dissemination policy	31a	Plans for investigators and sponsor to communicate trial results to participants, healthcare professionals, the public, and other relevant groups (eg, via publication, reporting in results databases, or other data sharing arrangements), including any publication restrictions
	31b	Authorship eligibility guidelines and any intended use of professional writers
	31c	Plans, if any, for granting public access to the full protocol, participant-level dataset, and statistical code
**Appendices**		

For peer review only

Informed consent materials	32	Model consent form and other related documentation given to participants and authorised surrogates
Biological specimens	33	Plans for collection, laboratory evaluation, and storage of biological specimens for genetic or molecular analysis in the current trial and for future use in ancillary studies, if applicable

*It is strongly recommended that this checklist be read in conjunction with the SPIRIT 2013 Explanation & Elaboration for important clarification on the items. Amendments to the protocol should be tracked and dated. The SPIRIT checklist is copyrighted by the SPIRIT Group under the Creative Commons "Attribution-NonCommercial-NoDerivs 3.0 Unported" license.

For peer review only

Preventing PTSD, depression, and associated health problems in student paramedics: Protocol for PREVENT-PTSD, a randomised controlled trial of supported online cognitive training for resilience versus alternative online training and standard practice

Journal:	*BMJ Open*
Manuscript ID	bmjopen-2018-022292.R1
Article Type:	Protocol
Date Submitted by the Author:	27-Jul-2018
Complete List of Authors:	Wild, Jennifer; University of Oxford, Experimental Psychology<br>El-Salahi, Shama; University of Oxford, Experimental Psychology<br>Tyson, Gabriella; University of Oxford, Experimental Psychology<br>Lorenz, Hjördis; University of Oxford, Experimental Psychology<br>Pariante, Carmine; King's College London, Psychological Medicine<br>Danese, Andrea; King's College London<br>Tsiachristas, Apostolos; University of Oxford, Health Economics Research Centre<br>Middleton, Benita; University of Surrey, Sleep, Chronobiology and Addiction<br>Blaber, Amanda; University of Brighton, School of Health Sciences<br>Ehlers, Anke; University of Oxford,
**Primary Subject Heading**:	Mental health
Secondary Subject Heading:	Immunology (including allergy), Health economics
Keywords:	Adult psychiatry < PSYCHIATRY, Anxiety disorders < PSYCHIATRY, Depression & mood disorders < PSYCHIATRY

Preventing PTSD, depression, and associated health problems in student paramedics: Protocol for PREVENT-PTSD, a randomised controlled trial of supported online cognitive training for resilience versus alternative online training and standard practice

Jennifer Wild[1,2], Shama El-Salahi[1], Gabriella Tyson[1], Hjördis Lorenz[1], Carmine M. Pariante[3], Andrea Danese[4,5], Apostolos Tsiachristas[6], Edward Watkins[7], Benita Middleton[8], Amanda Blaber[9], and Anke Ehlers[1,2]

[1] Oxford Centre for Anxiety Disorders and Trauma, Department of Experimental Psychology, University of Oxford, Oxford, UK
[2] Oxford Health NHS Foundation Trust
[3] Department of Psychological Medicine, Institute of Psychiatry, Psychology and Neuroscience, King's College London, London, UK
[4] Social, Genetic and Developmental Psychiatry, , Institute of Psychiatry, Psychology and Neuroscience, King's College London, London, UK
[5] Department of Child & Adolescent Psychiatry, Institute of Psychiatry, Psychology and Neuroscience, King's College London
[6] Health Economics Research Centre, Nuffield Department of Population Health, University of Oxford, Oxford, UK
[7]Sir Henry Wellcome Building for Mood Disorders Research, School of Psychology College of Life and Environmental Sciences, University of Exeter, Exeter, UK
[8] Faculty of Health and Medical Sciences, University of Surrey, Guildford, Surrey, UK
[9] School of Health Sciences, University of Brighton, Brighton, UK

Correspondence to: Dr Jennifer Wild; Oxford Centre for Anxiety Disorders and Trauma, The Old Rectory, Paradise Square, Oxford, OX1 1TW, 01865 618614, jennifer.wild@psy.ox.ac.uk

Word count: 5,946

Supplementary Files:

1. **Appendix 1. World Health Organization Trial Registration Data Set.**

For peer review only

2. Appendix 2. SPIRIT Checklist

3. Appendix 3. Participant Information Sheet and Consent Form.

Abstract

Introduction: Emergency workers dedicate their lives to promoting public health and safety, yet suffer higher rates of post-traumatic stress disorder (PTSD) and major depression (MD) compared to the general population. They also suffer an associated increased risk for physical health problems, which may be linked to specific immunological and endocrine markers or changes in relevant markers. Poor physical and mental health is costly to organisations, the NHS and society. Existing interventions aimed at reducing risk of mental ill health in this population are not very successful. More effective preventative interventions are urgently needed. We first conducted a large-scale prospective study of newly recruited student paramedics, identifying two cognitive factors (rumination and resilience appraisals) that predicted episodes of PTSD and MD over a two-year period. We then developed internet-delivered cognitive training for resilience (iCT-R), a supported online intervention, to modify cognitive predictors. This protocol is for a randomised controlled trial to evaluate the efficacy of the resilience intervention.

Methods and analysis: 570 student paramedics will be recruited from participating universities. They will be randomly allocated to iCT-R or to supported online training of an alternative, widely available intervention, or to training-as-usual. Follow-up will occur after the intervention/standard practice period, and at six, 12 and 24 months. Primary outcomes include rates of PTSD and MD and subsydnromal PTSD and MD,

For peer review only - http://bmjopen.bmj.com/site/about/guidelines.xhtml

measured by the Structured Clinical Interview for DSM-V (SCID-5), the Patient-Health Questionnaire-9 (PHQ-9) and the Posttraumatic Stress Disorder Checklist for DSM-5 (PCL-5). Secondary outcomes include measures of resilience, rumination, anxiety, psychological distress, wellbeing, salivary cortisol, plasma levels of C-reactive protein, smoking and alcohol use, weight gain, sleep problems, health-related quality of life, health resource utilization and productivity.

Ethics and dissemination: The Medical Sciences Inter-Divisional Research Ethics Committee at the University of Oxford granted approval, reference: R44116/RE001. The results will be published in a peer-reviewed journal. Access to raw data and participant information will be available only to members of the research team.

Trial registration number: ISRCTN16493616.

Keywords: student paramedics, PTSD, depression, resilience, cortisol, CRP

Strengths and limitations of this study

- The study is a large, single-blind randomised controlled trial of internet-delivered cognitive training for resilience (iCT-R).
- iCT-R will be evaluated in comparison to an existing intervention and treatment as usual.
- Primary outcomes will be assessed by self-report and objective measures.
- Full outcome blinding is not possible.
- Smoking and alcohol use will be measured with unpublished self-report tools.

For peer review only

Introduction

Emergency workers carry a threefold increase, compared to the general population, in risk for major depression (MD) and posttraumatic stress disorder (PTSD), and an associated increased risk of poor physical health.[1] To date, interventions aimed at reducing risk of ill mental health in this population have been unsuccessful. Randomised controlled trials (RCT) found that trauma risk management, a peer support system widely available to the police and ambulance services in England; [2] critical incident stress debriefing widely used by UK fire-services, [3] and the charity Mind's six-session group-based resilience intervention had no effect on resilience or rates of mental ill health [4]. More effective preventative interventions for emergency workers are urgently needed.

Established interventions may have been unsuccessful because they fail to target predictors of mental ill health and are offered to emergency workers after rather than before repeated exposure to the stresses linked to their work. Moreover, cognitive strategies that could help them cope with characteristic stressors are not included as part of the training. For example, our and others' research has demonstrated that exposure to trauma or stressful scenarios through imagery reduces anxiety for police officers and other at risk populations. [5, 6] Development of more effective interventions requires identification of predictors of mental disorders and an understanding of how to modify them.

For peer review only - http://bmjopen.bmj.com/site/about/guidelines.xhtml

In a series of experimental and prospective studies, we identified two cognitive factors that are robust predictors of poor mental health in emergency workers: rumination (repetitive negative thinking) and resilience appraisals. Those who reported ruminative thoughts during critical incidents were more likely to experience poor levels of coping. [7] Adaptive appraisals during analogue trauma led to more successful attempts to regulate emotions and fewer PTSD symptoms. [8] Our large-scale prospective study of newly recruited paramedics investigated predictors of PTSD and MD derived from cognitive theories of PTSD and depression. [1] Rumination at the start of paramedic training uniquely predicted PTSD; low resilience uniquely predicted an episode of MD.

We then developed an intervention to modify peri-traumatic ruminative thinking (i.e, thinking repetitively in an abstract way during trauma). Training to think in a concrete style (e.g., focusing on objective details and the sequence of events) led to significantly fewer intrusive memories and PTSD symptoms than individuals trained in a ruminative style. [9] We also applied one of the core techniques of a successful treatment for PTSD (cognitive therapy for PTSD,[10]), updating the memory of the stressful event with helpful information, as a preventative strategy for dealing with analogue trauma and found that it is more helpful in reducing repetitive thinking and PTSD symptoms than control interventions including exposure. [11]

Research has further demonstrated that exposure to trauma or stressful scenarios through imagery reduces anxiety for police officers and other at risk populations,

For peer review only - http://bmjopen.bmj.com/site/about/guidelines.xhtml

and that internet-based cognitive treatment that includes attention training as a core component significantly reduces anxiety. [5, 6, 12]

Neurobiological Factors linked to PTSD and MD

Genetic and longitudinal studies suggest that inflammation is a pre-existing vulnerability factor for the development of PTSD in trauma-exposed individuals rather than simply a correlate of subjective distress, disease severity, or maladaptive coping strategies following PTSD onset. [13, 14] For example, brain imaging studies have shown that high inflammation levels may increase threat perception (negative valence). Peripheral administration of lipopolysaccharides (LPS), residues from bacterial cells' components known to elicit a strong systemic inflammatory response, potentiates amygdala activity in response to socially threatening stimuli (fear faces).[15] In turn, greater pre-treatment amygdala reactivity to threat predicts less symptom reduction during CBT.[16] Additionally, inflammation is an important risk factor for depression and cardiovascular disease, which frequently accompany PTSD.[17-19] Our study will investigate the link between inflammation and the development of PTSD and MD in trauma-exposed student paramedics. We will investigate whether or not iCT-R can reduce levels of clinically-relevant inflammation levels, such as C-Reactive Protein (CRP), known to increase risk of psychiatric as well as cardiovascular and metabolic conditions comorbid with PTSD and MD.

Given the wealth of literature supporting a relationship between the stress hormone, cortisol, and PTSD and MD, we will also systematically assess the cortisol

For peer review only - http://bmjopen.bmj.com/site/about/guidelines.xhtml

awakening response (CAR) and diurnal cycle. The CAR is an endocrine marker, defined as the change in cortisol concentration that occurs during the first hour after waking from sleep.[20] A meta-analysis of 62 studies concluded that increases in the CAR were associated with job stress and life stress and linked to greater fatigue, burnout and exhaustion and risk for later health states, such as coronary heart disease.[21] A recent study found that higher CAR predicted future episodes of MD within a 2.5-year period.[20] We anticipate that iCT-R will reduce the CAR and cortisol throughout the day and protect against the development of PTSD and MD.

Study Objectives

The primary aim of the study is to evaluate the efficacy of internet cognitive training for resilience (iCT-R). We hypothesise that iCT-R will lead to fewer cases of PTSD and major depression (including subsyndromal PTSD and MD) and less severe PTSD and MD symptomatology at follow-up compared to an existing online training (Mind-Online) and standard practice.

Secondary Objectives

We hypothesise that iCT-R will lead to greater improvement in secondary outcome measures (resilience, rumination, hormone and immune function, smoking, weight gain, alcohol use, symptoms of anxiety, and sleep problems, psychological distress, wellbeing) than Mind-Online and standard practice. We also expect that iCT-R will be more cost-effective than Mind-Online and standard practice because of lower cost per participant without an episode or with low symptoms of PTSD or MD and

For peer review only - http://bmjopen.bmj.com/site/about/guidelines.xhtml

lower costs per quality adjusted life years (QALY) gained for participants receiving iCT-R.

Tertiary Objectives

We want to establish which baseline factors influence the effect of the interventions on primary and secondary outcomes so that we may make inferences about mechanisms of intervention efficacy. Understanding the effects that modifying risk and protective factors have may drive the refinement of future interventions. Our tertiary objectives are to determine which psychiatric, personality, trauma and social support factors at baseline (social support, trauma exposure, anxiety, age, gender, education, neuroticism, past and current psychiatric status, immune function) may influence (i.e., moderate) the effect of the interventions on levels of symptoms (PTSD or MD), psychological distress and wellbeing at follow-up. Determining which factors moderate outcome may inform improvements to the intervention. For example, should baseline factors, such as education or age moderate outcome, then the intervention could be improved in light of relevant moderators. This could include making it more accessible to younger participants with less education should this be relevant, for example. We will also investigate whether or not changes in resilience-related factors (rumination, responses to intrusions, concrete thinking, resilience appraisals, practice of iCT-R/Mind-Online tools) mediate symptom levels of PTSD and MD at one and two-year follow-up with iCT-R and Mind-Online. Finally, we will investigate whether or not concrete thinking, practice of tools and responses to

For peer review only - http://bmjopen.bmj.com/site/about/guidelines.xhtml

intrusions at 6 months predict diagnoses and levels of PTSD and depression symptoms at one and two-year follow-up.

Methods

The protocol includes all details required for the World Health Organization Trial Registration Data Set (Appendix 1) and was written in line with the SPIRIT Statement, which outlines recommendations for a minimum set of scientific, ethical, and administrative elements that should be addressed in a clinical trial (Appendix 2).[22]

Design

The proposed study is a single-blind (assessors blinded) randomised controlled trial in which N=570 student paramedics will be randomly allocated to receive internetdelivered cognitive training for resilience (iCT-R), an already available intervention (Mind Online) that has been investigated in previous trials or standard practice. Participants are also invited to give salivary and plasma samples before and after the interventions and at one and two-year follow-up. The trial will take place from

October 2017 to January 2021.

Participants

Student paramedics will be recruited from collaborating paramedic training programmes (University of Brighton, Oxford Brookes University, Bournemouth University, University of Hertfordshire, University of Worcester, University of Surrey

For peer review only - http://bmjopen.bmj.com/site/about/guidelines.xhtml

and Anglia Ruskin University). The locations selected constitute rural and city locations to improve generalisability. The researchers will present the study to each year group at collaborating universities to ensure the maximum reach of recruitment. After presenting the study, researchers will collect names and email addresses of interested students and email the registration survey including the participant information sheet (Appendix 3).

Inclusion and Exclusion Criteria

Students who are aged 18 and above, are training to be paramedics, and are in years 1, 2 or 3 of their paramedic training programme will be eligible for the study. They will be screened for levels of PTSD and MD, and a trained research assistant will contact participants if they score in the clinical range on measures of PTSD or MD, or report suicidal ideation to evaluate whether they are eligible or need treatment (under JW's supervision). The screening survey will trigger automatic notifications to the research assistant and the principal investigator if a participant scores 10 or above on the Patient Health Questionnaire 9 (PHQ-9)[23] or 1 or above on the suicidal ideation item of the same questionnaire or 33 or above on the Posttraumatic Stress Disorder Checklist for DSM-5 (PCL-5) [24]. Participants will be excluded from the study if their symptoms are interfering with their lives and they would like treatment, and the research assistant will offer them information on how to access evidence-based treatment for these conditions in local services.

For peer review only - http://bmjopen.bmj.com/site/about/guidelines.xhtml

For peer review only

Sample Size Calculation

The risk of student paramedics developing full syndromal PTSD and MD over two years without intervention is 10%, and 25% if subsyndromal PTSD and MD are included [1]. Since there are no interventions for emergency workers which target modifiable risk factors, we referred to a study with a similar approach to facilitate the calculation of power. Topper at al. (2017) evaluated the effects of an intervention targeting rumination on rates of depression in adolescents at one year follow-up in comparison to a waitlist condition.[25] The intervention reduced the rates of depression by 67% in comparison to the wait list condition. We estimated that our intervention, which also aims to modify rumination, would reduce rates of PTSD and depression by 50% in comparison to an existing intervention which has shown no change in rates of PTSD or MD over time. [4] Setting power at 80%, α=.05 and hypothesizing a reduction of relative risk of 50% gives an odds ratio of 0.429, which requires a total sample size of N=304 to show a risk reduction of 50% between iCT-R and the alternative intervention. Thus, each condition would require N=152. Since we have a third condition (standard practice), the total sample size required would be N=456. Allowing for a 20% rate of attrition, we will require a total sample size of N=570.

Randomisation and blinding

Participants will be randomised on a 1:1:1 ratio as per a computer-generated randomisation schedule stratified by site, gender and baseline PHQ-9 score (≥9 versus <9) and PCL-5 score (≥33 versus <33). The Oxford Clinical Trials Research Unit is independent to the research team and developed the randomisation programme.

For peer review only - http://bmjopen.bmj.com/site/about/guidelines.xhtml

The researchers will inform participants of the intervention they are to receive after they have completed baseline assessments. Outcome assessment will be single blind; questionnaires are completed online without any involvement of the researchers, the clinical interview will be conducted by an independent assessor blind to treatment allocation, and all personnel involved in processing and assessing the blood and saliva samples will be blinded to treatment allocation. Due to the nature of the interventions, participants cannot be completely blinded to allocation. However, the inclusion of an already available, alternative intervention aims to mitigate some risk of bias.

Intervention arms

Internet-delivered cognitive training for resilience (iCT-R) iCT-R aims to modify rumination and appraisals linked to low resilience in a sixsession supported online intervention. We include an imagery component, practice of strategies that been shown to prevent stress-related responses from developing,[8, 9] attention training,[12] and monthly top up exercises during followup to consolidate training, an approach that is lacking with existing interventions.

Our intervention follows a similar format to the internet-based programmes Clark, Ehlers, Wild and colleagues have developed for social anxiety and PTSD [12, 26]. The core information is delivered in six modules. The modules include whiteboard videos to explain concepts, audio files for practicing concrete thinking, testimonies from qualified paramedics and video footage of student paramedic call-outs for use in

For peer review only - http://bmjopen.bmj.com/site/about/guidelines.xhtml

experiential exercises. Following our findings of the protective benefits of concrete thinking [8] and the wealth of work in this area [i.e., 27] participants are regularly reminded to practise concrete thinking.

The modules are:

1. It Matters What you Focus On: Helpful and Unhelpful Attention
2. Get Out of Your Head with Helpful Thinking
3. Habits and Dwelling: How to Change Them
4. Dealing with Unwanted Memories: Then vs Now
5. Transforming Worries & Improving Performance
6. Beating Stress & Trauma: My Blueprint

A trained online coach (research assistant) provides email feedback on students' responses and, through an automated SMS programme, sends regular brief reminders of key points and notifications to practice IF-THEN plans (a technique shown to help individuals respond to warning signs for stress and dwelling).

Mind Online

The alternative intervention is a series of six modules available online covering information and advice about stress, sleep problems, anger, depression, PTSD and mindfulness. Participants will receive the same frequency, type and duration of remote support as in iCT-R.

Standard Practice

For peer review only - http://bmjopen.bmj.com/site/about/guidelines.xhtml

The third condition is training as usual. Participants will have access to the usual support offered through their university but they will not receive any online modules or remote support. They will be offered the iCT-R at the end of follow-up, when the study is completed.

Primary Outcome Measures

Levels of PTSD and MD

An independent assessor will administer the PTSD and MD modules of the Structured Clinical Interview for DSM-5 (SCID-5) to assess clinical and subsyndromal PTSD and MD [28]. The SCID-5 is an interview schedule for determining DSM-5 psychiatric diagnoses. PTSD and MD symptomatology will also be assessed with continuous measures: the PCL-5 and the PHQ-9 [24, 23], which will be completed at screening, which is typically the same day or shortly before the baseline questionnaires are released and completed. The PHQ-9 and PCL-5 scores at screening will be used as baseline scores in analyses. The PCL-5 is a self-report measure consisting of 20 questions that parallel the diagnostic criteria for PTSD set out in the Diagnostic and Statistical Manual of Mental Disorders, 5th edition [29]. Items are scored on a 5-point Likert scale (0 = “Not at all” to 4 = “Extremely”). The PHQ-9 is a well-validated 9-item self-report measure that assesses symptoms of depression. Items are scored on a 4-point Likert scale (0 = “Not at all” to 3 = “Nearly every day”). See Table 1 for the full list of outcomes, measures and assessment time points.

Table 1. Outcomes and measures

	Domain	Measures	Time point*

For peer review only - http://bmjopen.bmj.com/site/about/guidelines.xhtml

Primary Outcomes	PTSD	Structured Clinical Interview for DSM-5 Disorders (SCID-5).[28] Post-traumatic Stress Disorder Checklist (PCL-5).[24]	0 1 3 4<br>0 1 2 3 4
	MD	Structured Clinical Interview for DSM-V Disorders (SCID-5).[28]. Patient Health Questionnaire 9 (PHQ-9).[23]	0 1 3 4<br>0 1 2 3 4
Secondary Outcomes	Resilience	Connor-Davidson Resilience Questionnaire (CD-RISC).[30] Wagnild Resilience Scale.[31]	0 1 3 4<br>0 1 3 4
	Rumination	Ruminative Responses Scale (RRS) brooding subscale.[32]<br>Responses to Intrusions Questionnaire (RIQ) dwelling subscale.[33]	0 1 3 4
	Anxiety	Generalized Anxiety Disorder 7-item scale (GAD-7).[34]	0 1 3 4
	Smoking and Alcohol use	Smoking Behaviour Questionnaire.[1] Alcohol Use Questionnaire.[1]	0 1 3 4
	Weight and Height	Questionnaire recording participants' height and weight.[1]	0 1 3 4
	Psychological distress	General Health Questionnaire (GHQ-12).[35]	0 1 3 4
	Wellbeing	Warwick Edinburgh Mental Wellbeing Scale (WEMWBS).[36]	0 1 3 4
	Hormone function	Level of cortisol in response to awakening and throughout the day.	0 1 3 4
	Immune function	Level of C-reactive protein.	0 1 3 4
	Sleep problems	Insomnia Severity Index (ISI)[37]	0 1 3 4
	Health economics	EuroQol (EQ-5D-5L).[38]<br>Trimbos/iMTA Questionnaire for Costs Associated with Psychiatric Illness (TiC-P).[39]<br>Client Service Receipt Inventory.[40]<br>Health and Labor Questionnaire [41]	0 1 3 4<br>0 1 3 4<br><br>0 1 3 4<br>0 1 3 4
Tertiary Outcomes	Neuroticism	Eysenck Personality Questionnaire (EPQ) neuroticism subscale.[42]	0 1 3 4
	Social support	Social Support scale (SS) adapted from a brief measure of social support.[43]	0 1 3 4
	Demographics	General information questionnaire.[1]	0 1 3 4
	Trauma exposure	Trauma Screener.[44]	0 1 2 3 4
	Concrete thinking	Concrete thinking questionnaire, adapted from a previous concrete thinking assessment.[45]	0 1 2 3 4
	Intrusions	Duration, frequency and distress linked to Intrusions Questionnaire.[46]	0 1 2 3 4

*Timepoint: 0 = baseline, 1 = post-intervention, 2 = 6 month follow-up, 3 = 12 month follow-up, 4 = 24 month follow-up

Secondary Outcomes

Psychological outcomes

For peer review only - http://bmjopen.bmj.com/site/about/guidelines.xhtml

Two measures of resilience will be administered: the Wagnild Resilience Scale and the Connor-Davidson Resilience Questionnaire (CD-RISC). [31, 30] Two measures of resilience will be used since it is unclear which one most sensitively measures resilience in student paramedics. The Wagnild Resilience Scale is a 25-item scale that measures resilience by rating responses to statements on a 7-point Likert scale (0 = “Strongly disagree” to 7 = “Strongly agree”). The CD-RISC is a commonly used selfreport measure of resilience with 25 items scored on a 5-point Likert scale (0 = “Not true at all” to 4 = “True nearly all the time”). Both scales are well-validated measures with excellent psychometric properties. Rumination will be assessed with the brooding subscale of the Ruminative Responses Scale (RRS), a reliable and valid measure of the frequency of engaging in dwelling [32]. Rumination in response to unwanted memories will be assessed with the dwelling subscale of the Responses to Intrusions Questionnaire (RIQ), a reliable and valid measure of maladaptive responses to intrusive memories [33]. Anxiety will be measured with the Generalized Anxiety Disorder scale 7 (GAD-7), a 7-item scale with items scored on a 4-point Likert scale (0 = “Not at all” to 3 = “Nearly every day”) [34]. Psychological distress will be measured with the reliable and valid 12-item General Health Questionnaire 12 (GHQ-12) [35]. Wellbeing will be assessed using the Warwick Edinburgh Mental Wellbeing Scale (WEMWBS) [36]. The WEMWBS has 14 items and is scored on a 5-point Likert scale (1= “None of the time” to 5 = “All of the time”).

Hormone and immune function

For peer review only - http://bmjopen.bmj.com/site/about/guidelines.xhtml

Salivary cortisol will be assessed by RIA assay analysis. A sample of saliva will be collected upon awakening, 15, 30 and 60 minutes after awakening, and at 12 noon and 8pm. Baseline high-sensitive CRP plasma levels will be measured using an ILab 600 spectrophotometric method in serum samples.

Health outcomes

Smoking and alcohol use will be measured with unpublished questionnaires since two of our assessment points (1 and 2 year follow-up) require participants to report current use as well as changes in alcohol use and smoking over the previous year, a time period not currently referenced in validated tools. Shorter questionnaires may reduce response burden and improve questionnaire completion. Participants will be asked to indicate whether or not they smoke, how many cigarettes they smoke a day, and whether this has increased, decreased, or stayed the same in the last year. They will also be asked how many units of alcohol they have had in the last week, whether this is an average amount for them, and if not, how many units they usually drink per week. Weight gain will be measured by increases in BMI. Participants will be asked to provide their weight and height. The researchers will take weighing scales and a tape measure to study visits to weigh and measure participants. Sleep problems will be assessed by the Insomnia Severity Index (ISI), which is a reliable and valid brief self-report instrument of sleep quality and sleep difficulties.[37] In line with NICE guidelines, health related quality of life will be measured by the five-levels version of the EuroQol 5 Dimensions questionnaire (EQ-5D-5L).[38] The EQ-5D-5L is a validated and widely used generic measurement of health related quality of life based on five dimensions: mobility, self-care, usual activities, pain/discomfort, and

For peer review only - http://bmjopen.bmj.com/site/about/guidelines.xhtml

anxiety/depression. Although, we do not expect that all five dimensions will be affected by the intervention, we need to collect data on all dimensions in order to determine quality adjusted life years (QALYs).

Costs

The economic evaluation will be conducted by taking the NHS perspective (including the costs of using mental health services) and the broader societal perspective (including the costs of productivity loss due to illness). Mental health resource utilisation will be measured with an adapted version of the Clinical Service Receipt Inventory (CSRI) and valued by using published unit costs (e.g. NHS Reference Costs and Unit Costs of Health and Social Care).[40]. Productivity loss will be measured with Short Form-Health and Labor Questionnaire (SF-HLQ),[41] a validated questionnaire part of the Trimbos/iMTA questionnaire for Costs associated with Psychiatric Illness (TiC-P).[39]

Tertiary Outcomes

To assess potential moderators of outcomes, we will measure psychiatric, personality, trauma and social support factors at baseline (social support, trauma exposure, anxiety, age, gender, education, neuroticism, past and current psychiatric status, immune function). The neuroticism subscale (12 items) of the Eysenck Personality Questionnaire (EPQ) has excellent psychometric properties and is a measure of emotionality [42]. We will use an adapted version of a brief measure of social support (SS), to assess perceived support from and closeness to friends, family and work colleagues, as well as use of social support [43]. Trauma exposure will be

For peer review only

measured using a 19-item unpublished trauma questionnaire relevant to emergency workers, which includes items from the Life Events Checklist [44]. We will also collect demographic information (age, gender, and level of education), information on the duration, frequency and distress linked to the Intrusions Questionnaire [46], and questions about concrete and abstract thinking based on an existing assessment tool [45]. Participants will be asked to think about a problem they are having and write questions that may go through their minds in relation to the problem. They will then be presented with four problem scenarios and asked to select from a list the likely thoughts they would have if faced with the problem. The list consists of a range of concrete and abstract thoughts. We will investigate whether or not changes in resilience-related factors (rumination, responses to intrusions, concrete thinking, resilience appraisals, practice of iCT-R/Mind-Online tools) mediate symptom levels of PTSD and MD at one and two-year follow-up with iCT-R and Mind-Online. We will also investigate whether or not concrete thinking, practice of tools and responses to intrusions at 6 months predict diagnoses and levels of PTSD and depression symptoms at one and two-year follow-up.

Patient and Public Involvement

We held three User Advisory Groups with student paramedics who contributed to the design of the study, the selection of questionnaires, and the content of the intervention. The first User Advisory Group was co-organised with the Research Design Service South Central Patient and Public Involvement Officer, Megan BarlowPay. Research questions and outcome measures were discussed with all feedback incorporated, including the development of a module participants

For peer review only - http://bmjopen.bmj.com/site/about/guidelines.xhtml

requested to address socially anxious concerns common to their combined role as student and paramedic. A further two User Advisory Groups were held to review the intervention and to develop four versions of a questionnaire to assess concrete and abstract thinking in situations specific to student paramedics. Participants also completed the baseline questionnaires to assess their length of time and to provide feedback on the feasibility of administration. Participants were not involved in the recruitment and conduct of the study. Results will be made available in summary format to all participants by email once the study is completed.

Procedure

Researchers will present the study at collaborating universities and invite student paramedics to take part. Interested participants will be given a weblink to the study via our software platform, Qualtrics, where they can read and print a PDF copy of the Participant Information Sheet and discuss questions with the research assistant over the telephone. If they decide to take part, they will be emailed a link where they can login, re-read the Participant Information Sheet and complete a consent form (see Appendix 3 for the Participant Information Sheet and Consent Form). Written consent will be requested from participants before blood and saliva samples are taken. It will be made clear that participation is entirely voluntary and that volunteers may withdraw from the study at any point without incurring any negative consequences.

For peer review only - http://bmjopen.bmj.com/site/about/guidelines.xhtml

Participants will be recruited over a 12 month period. They will complete online two questionnaires (PCL-5 and PHQ-9) to assess eligibility. If participants score ≥10 on the PHQ-9, ≥33 on the PCL-5 or ≥1 on the PHQ-9 suicidal ideation item, a researcher will telephone the participant to determine symptom severity and whether treatment is necessary. A risk assessment will be conducted if a participant scores ≥1 on the PHQ-9 suicidal ideation item. If treatment is needed, participants will be signposted to their GP and local psychological services. If participants are eligible and consent to participate in the study, they will be assigned a participant ID number to ensure anonymity is maintained. They will complete a set of questionnaires online, take part in a clinical interview conducted by an independent assessor, and provide blood and saliva samples. The clinical interviews will be recorded on SanDisk MP3 recorders to ensure that the questions asked are standardised across all assessment interviews. Qualified phlebotomists will visit students at their universities to collect blood samples. During this study visit, participants will be provided with equipment to collect their saliva at home upon awakening and through the day (6 samples in total). Saliva and blood samples will be transported to the University of Surrey for assay analysis. The blood samples will be centrifuged immediately and only serum will be kept for analysis.

Once baseline assessments are completed, participants will be randomly allocated to iCT-R, Mind-Online or standard practice. When the interventions or standard practice are completed, participants will complete the full battery of assessments again; they will complete the questionnaires online, take part in a clinical interview,

For peer review only

and provide blood and saliva samples. Six months later, they will complete online a shorter set of questionnaires. At 12 and 24-month follow-up, they will complete the full battery of assessments again. See Figure 1 for a timeline of the study including the enrolment process, randomisation, interventions and assessments. We are aware that there are various tasks to complete at each assessment point. This may be offputting to participants and increase the likelihood of drop outs and respondent fatigue. We will clearly communicate the value of the assessments being administered, and participants will be compensated with £30 and a certificate of completion at follow up time points to discourage drop out. The reasons for nonadherence to the intervention or dropping out of the study will be recorded. An independent rater will assess treatment fidelity. The content of a random sample of email communications will be scored for reference to content relevant to each training programme.

Insert Fig 1. Study timeline.

Data Management

Registration and assessment data will be captured online via Qualtrics software. Participants will be assigned a unique code to be used for all data files and audio tapes. Access to the system will be restricted to named study personnel and via password protection. The University of Oxford's IT services have arranged for the files to be encrypted and backed up on a weekly basis using a Tivoli Storage Manager (TSM). The data will be copied to three separate tapes; one copy will reside in the Tape Robot in the IT Services Machine room and the other two are held in locked

For peer review only

For peer review only - http://bmjopen.bmj.com/site/about/guidelines.xhtml

fireproof safes, one onsite at IT services and one offsite in locked premises. The data on the tapes are inaccessible without the TSM database. The data on the offsite tapes are encrypted. Papers from clinical interviews will be kept in locked cabinets at the University of Oxford. The audiotapes from clinical interviews will be backed up online with password-protection and access restricted to study personnel. The blood samples will be centrifuged as soon as the laboratory at the University of Surrey receives them on the day of collection. The cellular component will be discarded and the serum will be stored at -80 °C. Saliva samples will be analysed at the University of Surrey by RIA assay analysis to detect levels of cortisol.

In line with the Oxford Clinical Trials Research Unit and the Medicines for Human Use Clinical Trials Regulations (2004), we have not recruited a Data Monitoring Committee (DMC) because recruitment and follow-up occur over a short period, there are minimal risks to participants and the trial protocol will not be modified regardless of the interim data.

Statistical Analyses

In line with the BMJ and Consort guidelines, data analysis will be intent-to-treat. All participants who have been randomised will be included in analyses, including those who drop out. We will compare dichotomous measures (rates of PTSD and MD, changes in alcohol use and smoking) between conditions using Chi square analysis. Continuous measures will be analysed using hierarchical linear modelling. This analysis models random slopes and intercepts for participants, and tests the fixed effects of repeated assessments over time (level 1, pre-intervention,

For peer review only - http://bmjopen.bmj.com/site/about/guidelines.xhtml

postintervention, 1 and 2 year follow-up) and training condition (level 2, iCT-R, MindOnline, Standard Practice) using data from all participants. It takes into account that participants are nested within site (level 3). Variables will be centred for the analysis. The effects of potential moderators (social support, exposure to critical incidents, etc.) on PTSD and depression symptoms will be explored by including main effects and interactions with treatment effects into the model. Non-significant moderators will be removed from the final model.

To address the potential for Type I error when evaluating our secondary outcomes (i.e., resilience, rumination, hormone and immune function, smoking, weight gain, alcohol use, anxiety, sleep problems, psychological distress, wellbeing), we will examine and report effect sizes. Effect sizes are a reliable method for determining the quality of the result that do not rely on p-value significance and are not affected by the number of outcomes.

Mediation analyses will be conducted to assess whether or not changes in resiliencerelated factors (rumination, responses to intrusions, concrete thinking, resilience appraisals, practice of iCT-R/Mind-Online tools) and compliance with the training programmes mediate symptom levels of PTSD and MD at one and two-year followup with iCT-R and Mind-Online.

A trial-based economic evaluation will be conducted to investigate the costeffectiveness of the intervention in terms of cost per QALY gained. Uncertainty

For peer review only - http://bmjopen.bmj.com/site/about/guidelines.xhtml

in the results will be addressed in sensitivity analyses and displayed in costeffectiveness planes and cost-effectiveness acceptability curves.

Adverse Events

We do not anticipate any adverse events. However, it is possible that a participant may evidence risk at one of the assessment points (pre-intervention, postintervention, or 12 and 24-month follow-up). If this is the case, risk will be assessed over the telephone and the individual will be signposted to the appropriate service.

Should a serious adverse event (SAE) occur where, in the opinion of the Principal Investigator, the event was 'related' (resulted from administration of any of the research procedures) and 'unexpected' in relation to those procedures, it will be reported to the Research Ethics Committee. Reports of related and unexpected SAEs will be submitted within 15 working days of the Principal Investigator becoming aware of the event, using the Health Research Authority safety report form for a non-Clinical Trial of an Investigation of a Medicinal Product (non-CTIMP).

The University of Oxford has a specialist insurance policy in place, which would operate in the event of any participant suffering harm as a result of their involvement in the research (Newline Underwriting Management Ltd, at Lloyd's of London).

For peer review only - http://bmjopen.bmj.com/site/about/guidelines.xhtml

ETHICS AND DISSEMINATION

Ethical approval of the research protocol was gained from The Medical Sciences Inter-Divisional Research Ethics Committee at the University of Oxford, 17/08/2017, ref: R44116/RE001. This is protocol version 1. Any substantive amendments to the protocol will be conducted by the Principal Investigator and reviewed by the Research Ethics Committee.

The research results will be submitted for publication in a peer-reviewed journal and presented at relevant conferences. Direct access to data will be granted to authorised representatives from the host institution and the regulatory authorities to permit trial-related monitoring, audits and inspections.

Committees. We have established a Trial Oversight Committee (TOC). Our independent chairman is Dr Susan Dutton, the Senior Medical Statistician and Oxford Clinical Trials Research Unit Lead Statistician. The Principal Investigator is also a member of the TOC and we have one lay qualified paramedic member (Graham Harris). The TOC will meet before the start of the trial and three more times before the end of the trial.

Acknowledgements. We are grateful to Dr Esther Beierl for conducting the independent statistical analyses for this study, and to Dr Susan Dutton for chairing the Trial Oversight Committee. We are grateful to Megan Barlow-Pay for facilitating the first User Advisory Group. We thank our User Advisory Group members, who

For peer review only - http://bmjopen.bmj.com/site/about/guidelines.xhtml

have contributed to the development of the intervention and the design of the study.

Contributors. All authors contributed substantially to conception and the design of the protocol or the acquisition of data for the work. All revised the manuscript critically for important intellectual content, all approved the final manuscript, and all are accountable for all aspects of the work in ensuring that questions related to the accuracy or integrity of any part of the work are appropriately investigated and resolved.

JW is the Principal Investigator and will oversee the study and arrange and lead meetings with a User Advisory Group and the Trial Oversight Committee. JW conceived and co-designed the study with AE. JW developed the intervention with AE, EW, and GT. JW completed the ethics application, and will liaise with collaborators to support recruitment, manage and supervise the research team and write the primary paper for publication. AB will facilitate recruitment and contact with collaborating centres. GT will offer online support to participants receiving the online interventions and help to schedule assessments. SE will be the independent assessor at baseline and follow-up. JW, GT, SE and HL are responsible for data collection. BM will analyse the biological data. CP, AD and AT will guide the analyses of the biological and health economics data.

Funding. This work is funded by an MQ: Transforming Mental Health grant (number

For peer review only

CQR01260) and supported by the NIHR Oxford Health Biomedical Research Centre. MQ had no role in the design of this study and will not have any role during its execution, analyses, interpretation of the data, or decision to submit results. Anke Ehlers is funded by a Wellcome Trust Principal Research Fellowship (grant 200796). CMP is supported by the NIHR Biomedical Research Centre at the South London and Maudsley NHS Trust and King's College London, London, UK. The views expressed are those of the authors and not necessarily those of the NHS, the NIHR or the Department of Health.

Competing interests. Dr Jennifer Wild, Professor Anke Ehlers and their team have developed iCT-R. They do not receive any income from this work.

For peer review only

References

1 Wild J, Smith KV, Thompson E, et al. A prospective study of pre-trauma risk factors or posttraumatic stress disorder and depression. *Psychol Med* 2016;46(1):2571-82.

2 Greenberg N, Langston V, Everitt, B., Iversen, A., Fear, N.T., Jones, N. & Wessely, S. (2010). A cluster randomised controlled trial to determine the efficacy of trauma risk management (TRiM) in a military population. *J Trauma Stress, 23,* 430-36.

3 van Emmerik AA, Kamphuis JH, Hulsbosch AM, Emmelkamp PM (2002). Single session debriefing after psychological trauma: a meta-analysis. *Lancet* 2002;360:76671.

4 Wild, J. (2016). An evaluation of Mind's resilience intervention for emergency workers. Final Report.

5 Arnetz BB, Arble E, Backman L, Lynch A, & Lublin A. Assessment of a prevention program for work-related stress among urban police officers. *Int Arch Occup Environ Health* 2013;86:79-88.

6 Wild J, Hackmann A, Clark DM. Rescripting early memories linked to negative images in social phobia: A pilot study. *Behav Ther* 2008;39:47-56.

7 Shepherd L, Wild J. Cognitive appraisals, objectivity, and coping in ambulance workers: a pilot study. *Emerg Med J* 2013;31:41-4.

8 Shepherd L, Wild J. Emotion regulation, physiological arousal and PTSD symptoms in trauma-exposed individuals. *J Behav Ther Exp Psychiatry* 2014;45:360-7.

9 White R, Wild J. "Why" or "How": The effect of concrete versus abstract processing on intrusive memories following analogue trauma. *Behav Ther* 2016:47(3):404-415.

10 Ehlers A., Hackmann A., Grey N, Wild J, et al. A randomized controlled trial of 7day intensive and standard weekly cognitive therapy for PTSD and emotion-focused supportive therapy. *Am J Psychiatry,* 2014:171, 294-304.

11 Pile V, Barnhofer T, Wild J. Updating versus exposure to prevent consolidation of conditioned fear. *PLoS One* 2015;10:1-21.

For peer review only

12 Stott R, Wild J, Grey N et al. Internet-delivered cognitive therapy for social anxiety disorder: a development series. *Behav Cogn Psychother* 2014;41:383-97.

13 Michopoulos V, Rothbaum AO, Jovanovic T, et al. Association of CRP genetic variation and CRP level with elevated PTSD symptoms and physiological responses in a civilian population with high levels of trauma. *Am J Psychiatry,* 2015;172:353-362.

14 Eraly SA, Nievergelt CM, Maihofer AX, et al. Assessment of plasma C-reactive protein as a biomarker of posttraumatic stress disorder risk. *JAMA Psychiatry* 2014;71:423-431.

15 Inagaki TK, Keely A, Muscatell MA, et al. Inflammation Selectively Enhances Amygdala Activity to Socially Threatening Images. *Neuroimage 2012;*15:3222-6.

16 Bryant RA, Felmingham K, Kemp A, et al. Amygdala and ventral anterior cingulate activation predicts treatment response to cognitive behaviour therapy for posttraumatic stress disorder. *Psychol Med* 2008;*38:*555-61.

17 Miller AH, Raison CL. The role of inflammation in depression: from evolutionary imperative to modern treatment target. *Nat Rev in Immunol* 2016;16:22-34.

18 Ridker PM, Cushman M, Stampfer MJ, et al. Inflammation, Aspirin, and the Risk of Cardiovascular Disease in Apparently Healthy Men. *N Engl J Med* 1997;336:973-9.

19 Sumner JA, Kubzansky LD, Elkind MS, et al. Trauma Exposure and Posttraumatic Stress Disorder Symptoms Predict Onset of Cardiovascular Events in Women. *Circulation* 2015;8:251-9.

20 Vrshek-Schallhorn S, Doane LD, Mineka S, et al. The cortisol awakening response predicts major depression: predictive stability over a 4-year follow-up and effect of depression history. *Psychol Med* 2013;43:483-93.

21 Chida Y, Steptoe A. Cortisol awakening response and psychosocial factors: a systematic review and meta-analysis. *Biol Psychiatry* 2009;80:265-78.

22 Chan AW, Tetzlaff JM, Altman DG, et al. SPIRIT 2013 Statement: Defining standard protocol items for clinical trials. *Ann Intern Med* 2013;158:200-7.

23 Kroenke K, Spitzer R, Williams JB. The PHQ-9. Validity of a Brief Depression Severity Measure. *J Gen Intern Med* 2001;16:603-13.

24 Weathers FW, Litz BT, Keane TM, et al. *The PTSD Checklist for DSM-5 (PCL-5)* 2013. Scale available from the National Center for PTSD at www.ptsd.va.gov.

For peer review only

25 Topper M, Emmelkamp PM, Watkins E, Ehring T. Prevention of anxiety disorders and depression by targeting excessive worry and rumination in adolescents and young adults: A randomized controlled trial. *Behav Res Ther* 2017;90:123-136.

26 Wild J, Warnock-Parkes E, Grey N, et al. Internet-delivered cognitive therapy for PTSD: a development pilot series. *Eur J Psychotraumatol* 2016;7:10.3402/ejpt.v7.31019. doi:10.3402/ejpt.v7.31019.

27 Watkins E, Taylor RS, Byngs R, et al. Guided self-help concreteness training as an intervention for major depression in primary care: a Phase II randomized controlled trial. *Psychol Med* 2011;*42:*1359-71.

28 First MB, Williams JBW, Karg RS, et al. Structured Clinical Interview for DSM-5, Clinician Version (SCID-5-CV). Arlington, VA: American Psychiatric Association, 2016.

29 American Psychiatric Association. Diagnostic and statistical manual of mental disorders (5th ed.). Arlington, VA: American Psychiatric Publishing, 2013.

30 Connor KM, Davidson JRT. Development of a new resilience scale: the ConnorDavidson Resilience Scale (CD-RISC). *Depress Anxiety* 2003;18:76-82.

31 Wagnild GM, Young HM. Development and psychometric evaluation of the Resilience Scale. *J Nurs Meas* 1993;1(2):165-78.

32 Treynor W, Gonzalez R, Nolen-Hoeksema S. Rumination reconsidered: a psychometric analysis. *Cognit Ther Res* 2003;27:247-59.

33 Clohessy S, Ehlers A. PTSD symptoms, response to intrusive memories, and coping in ambulance service workers. *Br J Clin Psychol* 1999;38:251-65.

34 Spitzer RI, Kroenke K, Williams JW et al. A brief measure for assessing generalized anxiety disorder: The GAD-7. *Arch Intern Med* 2006;166(10):1092-7.

35 Goldberg D, Williams P. A user's guide to the General Health Questionnaire. Windsor, UK: NFER-Nelson 1988.

36 Tennant R, Hiller L, Fishwick R, et al. The Warwick-Edinburgh Mental Well-being Scale (WEMWBS): development and UK validation. *Health Qual Life Outcomes* 2007;5:63.

37 Morin CM, Belleville G, Bélinger L, et al. The Insomnia Severity Index: psychometric indicators to detect insomnia cases and evaluate treatment response. *Sleep* 2011;34(5):601-8.

For peer review only

38 EuroQol: the current state of play. *Health Policy* 1996;37:53-72.

39 Hakkaart-van Roijen L, Van Straten A, Donker M, et al. Manual Trimbos/iMTA questionnaire for Costs associated with Psychiatric Illness (TiC-P). Rotterdam: Institute for Medical Technology Assessment, Erasmus MC Rotterdam 2002.

40 Chisholm D, Knapp MR, Knudsen HC, et al. Client Socio-Demographic and Service Receipt Inventory--European Version: development of an instrument for international research. EPSILON Study 5. European Psychiatric Services: Inputs Linked to Outcome Domains and Needs. *Br J Psychiatry Suppl*. 2000(39):s28-33. 41 Roijen VL, Essink-Bot M, Koopmanschap M, et al. Labor and health status in economic evaluation of health care. The Health and Labor Questionnaire. *Int J Technol Assess Health Care* 1996;12(3):405-415.

42 Eysenck HJ, Eysenck SBG. Manual of the Eysenck Personality Questionnaire. London: Hodder & Stoughton 1975.

43 Sarason IG, Sarason BR, Shearin EN, et al. A brief measure of social support: Practical and Theoretical Implications. *J Soc Pers Relat* 1987;4:497-510.

44 Gray MJ, Litz BT, Hsu JL, Lombardo TW. Psychometric properties of the Life Events Checklist. *Assessment* 2004;11(4):330-341.

45 Stöber J, Borkovec TD. Reduced concreteness of worry in generalized anxiety disorder: Findings from a therapy study. *Cognit Ther Res* 2002;26:89–96.

46 Michael T, Ehlers A, Halligan SL, Clark DM. Unwanted memories of assault: what intrusion characteristics are associated with PTSD? *Behav Res Ther* 2005;43(5):613628.

FIGURE LEGENDS

Figure 1. Study timeline. The flowchart shows how participants will progress through the study from the initial stage of enrolment through to analysis.

For peer review only

Fig 1. Study timeline.

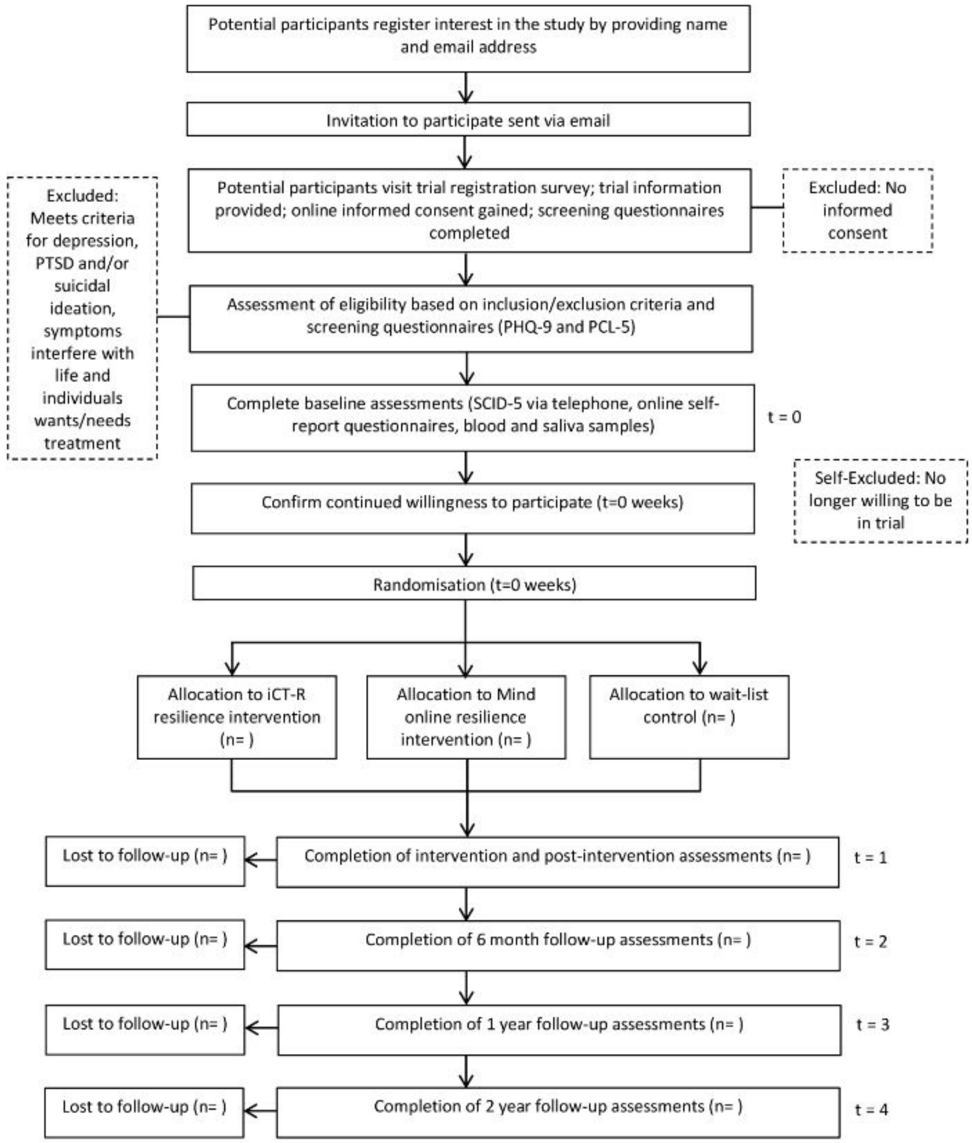

Figure 1. Study timeline. The flowchart shows how participants will progress through the study from the initial stage of enrolment through to analysis.

188x229mm (96 x 96 DPI)

Appendix 1. World Health Organization Trial Registration Data Set.

DATA CATEGORY	INFORMATION
Primary registry and trial identifying number	Registry: ISRCTN. Identifying number: ISRCTN16493616.
Date of registration in primary registry	9 October, 2017
Secondary identifying numbers	Protocol/serial number: PREVENT-PTSD/Protocol V1.
Source(s) of monetary or material support	MQ: Transforming Mental Health
Primary sponsor	MQ: Transforming Mental Health
Secondary sponsor(s)	University of Oxford
Contact for public queries	*JW* +44(0)1865 618612, jennifer.wild@psy.ox.ac.uk
Contact for scientific queries	*JW* +44(0)1865 618612, jennifer.wild@psy.ox.ac.uk
Public title	PREVENT-PTSD
Scientific title	Preventing PTSD, depression, and associated health problems in student paramedics: Protocol for PREVENT-PTSD, a randomised controlled trial of supported online cognitive training for resilience versus alternative online training and standard practice
Countries of recruitment	England
Health condition(s) or problem(s) studied	Post-traumatic stress disorder (PTSD), major depression (MD), resilience, adverse health behaviours (sleep problems, weight gain and smoking), hormone and immune function, utilization of health services and productivity loss.
Intervention(s)	Active comparator: Resilience intervention focusing on modifying rumination and cognitive appraisals<br>Control comparator: Online information and advice about stress, sleep problems, anger, depression PTSD and mindfulness<br>Standard practice comparator: Training as usual
Key inclusion and exclusion criteria	Ages eligible for study: ≥18 years<br>Sexes eligible for study: both<br>Accepts healthy volunteers: yes<br>Inclusion criteria: student paramedics (≥ 18 years) who do not currently have depression or PTSD needing treatment and who are not actively suicidal.<br>Exclusion criteria: student paramedics who meet criteria for depression or PTSD and whose lives are significantly impacted by their symptoms, thereby needing treatment; student paramedics who express suicidal ideation and intent.
Study type	Interventional<br>Allocation: randomised<br>Intervention model: parallel assignment<br>Masking: assessors blinded to all assessments<br>Primary purpose: prevention<br>Phase I
Date of first enrolment	October 2017
Target sample size	570

For peer review only

Recruitment status	Recruiting
Primary outcome(s)	Diagnoses of PTSD and MD assessed by an independent assessor using the Structured Clinical Interview for DSM-5
Key secondary outcomes	Mental Health: resilience, rumination, symptoms of anxiety, and sleep problems, psychological distress, wellbeing<br><br>Immune and Endocrine Function: cortisol awakening response and plasma levels of C-reactive protein<br><br>Physical health behaviours: smoking, weight gain, alcohol use.<br><br>Health Economics: utilisation of health services, episodes of PTSD and MD, absenteeism and presenteeism

For peer review only

Appendix 2

STANDARD PROTOCOL ITEMS: RECOMMENDATIONS FOR INTERVENTIONAL TRIALS

SPIRIT 2013 Checklist: Recommended items to address in a clinical trial protocol and related documents*

Section/item	Item No	Description	Addressed on page number
**Administrative information**			
Title	1	Descriptive title identifying the study design, population, interventions, and, if applicable, trial acronym	1
Trial registration	2a	Trial identifier and registry name. If not yet registered, name of intended registry	3
	2b	All items from the World Health Organization Trial Registration Data Set	Supplemental File Appendix 1
Protocol version	3	Date and version identifier	25
Funding	4	Sources and types of financial, material, and other support	26
Roles and responsibilities	5a	Names, affiliations, and roles of protocol contributors	26
	5b	Name and contact information for the trial sponsor	N/A
	5c	Role of study sponsor and funders, if any, in study design; collection, management, analysis, and interpretation of data; writing of the report; and the decision to submit the report for publication, including whether they will have ultimate authority over any of these activities	26
	5d	Composition, roles, and responsibilities of the coordinating centre, steering committee, endpoint adjudication committee, data management team, and other individuals or groups overseeing the trial, if applicable (see Item 21a for data monitoring committee)	25

For peer review only

**Introduction**			
Background and rationale	6a	Description of research question and justification for undertaking the trial, including summary of relevant studies (published and unpublished) examining benefits and harms for each intervention	4-7
	6b	Explanation for choice of comparators	7
Objectives	7	Specific objectives or hypotheses	7-9
Trial design	8	Description of trial design including type of trial (eg, parallel group, crossover, factorial, single group), allocation ratio, and framework (eg, superiority, equivalence, noninferiority, exploratory)	9
**Methods: Participants, interventions, and outcomes**			
Study setting	9	Description of study settings (eg, community clinic, academic hospital) and list of countries where data will be collected. Reference to where list of study sites can be obtained	9
Eligibility criteria	10	Inclusion and exclusion criteria for participants. If applicable, eligibility criteria for study centres and individuals who will perform the interventions (eg, surgeons, psychotherapists)	10
Interventions	11a	Interventions for each group with sufficient detail to allow replication, including how and when they will be administered	12-13
	11b	Criteria for discontinuing or modifying allocated interventions for a given trial participant (eg, drug dose change in response to harms, participant request, or improving/worsening disease)	N/A
	11c	Strategies to improve adherence to intervention protocols, and any procedures for monitoring adherence (eg, drug tablet return, laboratory tests)	21
	11d	Relevant concomitant care and interventions that are permitted or prohibited during the trial	N/A
Outcomes	12	Primary, secondary, and other outcomes, including the specific measurement variable (eg, systolic blood pressure), analysis metric (eg, change from baseline, final value, time to event), method of aggregation (eg, median, proportion), and time point for each outcome. Explanation of the clinical relevance of chosen efficacy and harm outcomes is strongly recommended	13-19
Participant timeline	13	Time schedule of enrolment, interventions (including any run-ins and washouts), assessments, and visits for participants. A schematic diagram is highly recommended (see Figure)	21

For peer review only - http://bmjopen.bmj.com/site/about/guidelines.xhtml

Sample size	14	Estimated number of participants needed to achieve study objectives and how it was determined, including clinical and statistical assumptions supporting any sample size calculations	10-11
Recruitment	15	Strategies for achieving adequate participant enrolment to reach target sample size	9

Methods: Assignment of interventions (for controlled trials)

Allocation:			
Sequence generation	16a	Method of generating the allocation sequence (eg, computer-generated random numbers), and list of any factors for stratification. To reduce predictability of a random sequence, details of any planned restriction (eg, blocking) should be provided in a separate document that is unavailable to those who enrol participants or assign interventions	11
Allocation concealment mechanism	16b	Mechanism of implementing the allocation sequence (eg, central telephone; sequentially numbered, opaque, sealed envelopes), describing any steps to conceal the sequence until interventions are assigned	11
Implementation	16c	Who will generate the allocation sequence, who will enrol participants, and who will assign participants to interventions	11
Blinding (masking)	17a	Who will be blinded after assignment to interventions (eg, trial participants, care providers, outcome assessors, data analysts), and how	11-12
	17b	If blinded, circumstances under which unblinding is permissible, and procedure for revealing a participant's allocated intervention during the trial	N/A

Methods: Data collection, management, and analysis

Data collection methods	18a	Plans for assessment and collection of outcome, baseline, and other trial data, including any related processes to promote data quality (eg, duplicate measurements, training of assessors) and a description of study instruments (eg, questionnaires, laboratory tests) along with their reliability and validity, if known. Reference to where data collection forms can be found, if not in the protocol	13-19
	18b	Plans to promote participant retention and complete follow-up, including list of any outcome data to be collected for participants who discontinue or deviate from intervention protocols	21

Data management	19	Plans for data entry, coding, security, and storage, including any related processes to promote data quality (eg, double data entry; range checks for data values). Reference to where details of data management procedures can be found, if not in the protocol	21-22
Statistical methods	20a	Statistical methods for analysing primary and secondary outcomes. Reference to where other details of the statistical analysis plan can be found, if not in the protocol	22-23
	20b	Methods for any additional analyses (eg, subgroup and adjusted analyses)	N/A
	20c	Definition of analysis population relating to protocol non-adherence (eg, as randomised analysis), and any statistical methods to handle missing data (eg, multiple imputation)	22
**Methods: Monitoring**			
Data monitoring	21a	Composition of data monitoring committee (DMC); summary of its role and reporting structure; statement of whether it is independent from the sponsor and competing interests; and reference to where further details about its charter can be found, if not in the protocol. Alternatively, an explanation of why a DMC is not needed	N/A
	21b	Description of any interim analyses and stopping guidelines, including who will have access to these interim results and make the final decision to terminate the trial	N/A
Harms	22	Plans for collecting, assessing, reporting, and managing solicited and spontaneously reported adverse events and other unintended effects of trial interventions or trial conduct	24
Auditing	23	Frequency and procedures for auditing trial conduct, if any, and whether the process will be independent from investigators and the sponsor	N/A
**Ethics and dissemination**			
Research ethics approval	24	Plans for seeking research ethics committee/institutional review board (REC/IRB) approval	25
Protocol amendments	25	Plans for communicating important protocol modifications (eg, changes to eligibility criteria, outcomes, analyses) to relevant parties (eg, investigators, REC/IRBs, trial participants, trial registries, journals, regulators)	N/A

Consent or assent	26a	Who will obtain informed consent or assent from potential trial participants or authorised surrogates, and how (see Item 32)	19
	26b	Additional consent provisions for collection and use of participant data and biological specimens in ancillary studies, if applicable	N/A
Confidentiality	27	How personal information about potential and enrolled participants will be collected, shared, and maintained in order to protect confidentiality before, during, and after the trial	21-22
Declaration of interests	28	Financial and other competing interests for principal investigators for the overall trial and each study site	26-27
Access to data	29	Statement of who will have access to the final trial dataset, and disclosure of contractual agreements that limit such access for investigators	25
Ancillary and post-trial care	30	Provisions, if any, for ancillary and post-trial care, and for compensation to those who suffer harm from trial participation	N/A
Dissemination policy	31a	Plans for investigators and sponsor to communicate trial results to participants, healthcare professionals, the public, and other relevant groups (eg, via publication, reporting in results databases, or other data sharing arrangements), including any publication restrictions	25
	31b	Authorship eligibility guidelines and any intended use of professional writers	N/A
	31c	Plans, if any, for granting public access to the full protocol, participant-level dataset, and statistical code	N/A
**Appendices**			
Informed consent materials	32	Model consent form and other related documentation given to participants and authorised surrogates	Supplementary File Appendix 3
Biological specimens	33	Plans for collection, laboratory evaluation, and storage of biological specimens for genetic or molecular analysis in the current trial and for future use in ancillary studies, if applicable	22

*It is strongly recommended that this checklist be read in conjunction with the SPIRIT 2013 Explanation & Elaboration for important clarification on the items. Amendments to the protocol should be tracked and dated. The SPIRIT checklist is copyrighted by the SPIRIT Group under the Creative Commons "Attribution-NonCommercial-NoDerivs 3.0 Unported" license.

For peer review only

Oxford Centre for Anxiety Disorders and Trauma
The Old Rectory
Paradise Square, Oxford
OX1 1TW

Dr Jennifer Wild
Jennifer.wild@psy.ox.ac.uk Tel: 01865 618 612
Gabriella Tyson (Research Assistant)
Oxford telephone number: 01865 618 610
Oxford email address: gabriella.tyson@psy.ox.ac.uk

A study of resilience training for student paramedics

PARTICIPANT INFORMATION SHEET

Ethics Approval Reference: R44116/RE001

1. *Background and aims of the study*

Research indicates that paramedics carry an increased risk for depression and a severe stress condition called posttraumatic stress disorder (PTSD) due to the nature of their work. Our past research identified early predictors of these problems in student paramedics. We have now developed a training programme that aims to prevent these problems from developing by modifying the predictors linked to their onset. The study hopes to answer the following questions:

Do student paramedics benefit from resilience training?
Which intervention, if any, best helps student paramedics?
Are the interventions associated with improvements in physical health?

This study is funded by MQ. The courses are free and will be delivered online with the support of a wellbeing coach.

2. *Why have I been invited to take part?*

You have been invited to take part in the study because you are a student paramedic between the ages of 18 and 65 years.

The inclusion criteria are students who are training to be paramedics and who are in years 1, 2 or 3 of their paramedic programme.

The exclusion criteria are students who score in the clinical range on screening measures of post-traumatic stress and depression and would also benefit from treatment since psychological treatment is likely to be more helpful.

3. *Do I have to take part?*

No. You can ask questions about the study before deciding whether or not to participate. If you do agree to participate, you may withdraw yourself and your data from the study at any time, without giving a reason and without penalty, by advising the researchers of this decision.

4. *What will happen in the study?*

If you are happy to take part in the study, you will be asked to fill in two short questionnaires about depression and anxiety. You will not be able to take part if these questionnaires suggest that you may have

For peer review only - http://bmjopen.bmj.com/site/about/guidelines.xhtml

one of these problems and would also benefit from treatment. If this is the case, the researcher will talk with you and give you suggestions about what may be helpful. This could be a visit to your GP or accessing other local services or both. The screening questionnaires will be destroyed after use.

You will be able to take part if the questionnaires suggest that you do not have depression or post-traumatic stress.

You will be invited to complete a longer set of questionnaires that measure levels of wellbeing, resilience and stress and to answer a few questions about stress symptoms over the telephone with our research assistant at a time that is convenient to you. Once you have completed these, you will be randomly allocated to one of the two internet-based courses which will start within a few weeks or to standard practice, which means you would receive an internet-based course at the end of two years. 70% of the people in the study will be able to start the course right away. The remaining 30% will be offered the course after two years. **The decision about which intervention you will receive will be made by chance.**

You will be invited to give a blood sample (1 teaspoon) before the course (or standard practice), immediately after, 12 and 24 months post course (or standard practice). Blood samples will be taken by trained staff. Samples will be analysed for a marker of inflammation called C-reactive protein. No cellular constituents will be stored or analysed.

You will also be invited to take 6 samples of your saliva (to measure cortisol, a stress hormone) upon awakening, 15, 30 and 60 minutes after awakening, and at 12 noon and 8 pm. Full instructions will be given on how to do this in your home. You will be provided with a Royal Mail Safebox to securely post the samples to the University of Surrey where they will be analysed.

The main phase of the course is 6 weeks. If you are allocated to either of the internet-based courses you will work through the internet programme modules in the comfort of your home with support from a wellbeing coach via SMS or email, depending on your preference. The internet programme will require you to dedicate up to an hour a week in the first 6 weeks of the course, and after that you will receive regular reminders and top up exercises.

Over the course of the interventions, we will ask you to complete questionnaires at five time points: before the intervention, after the intervention, six, 12 and 24 months after the intervention. The questionnaires take 20 minutes to complete at all time points except at 6 months post-intervention, when they will take just 10 minutes to complete. Therefore 105 minutes in the first year and 80 minutes in the second year would be required to complete questionnaires and telephone interviews. The timing of visits and what would be done at each visit is below:

Baseline
At home – two online questionnaires (10 minutes)

1 to 7 days later
At home – online questionnaires (20 minutes) + telephone interview (15 minutes) + saliva samples (10 minutes)
Visit 1 at university – Blood sample with our phlebotomist

6 weeks later
At home – online questionnaires (20 minutes) + saliva samples (10 minutes) + telephone interview (10 minutes)
Visit 2 at university - Blood sample with our phlebotomist

24 weeks later
At home – online questionnaires (10 minutes)

12 months later
At home – online questionnaires (20 minutes) + saliva samples (10 minutes) + telephone interview (15

For peer review only - http://bmjopen.bmj.com/site/about/guidelines.xhtml

minutes)
Visit 3 at university - Blood sample with our phlebotomist

24 months later
At home – online questionnaires (20 minutes) + saliva samples (10 minutes) + telephone interview (15 minutes)
Visit 4 at university - Blood sample with our phlebotomist

The telephone interviews will be audio-recorded. This is so that at a later date an independent assessor can rate a random sample of audio-recordings to ensure that the interviews have followed the study protocol. This process refers to quality assurance. The audio files are confidential and will be securely transferred to the desktop of the principal researcher at the University of Oxford. All audio files will be deleted within 7 years of the study ending.

5. ***Are there any potential risks in taking part?***

There are no risks associated with completing the questionnaires or the interventions or receiving standard practice or taking saliva samples. There are common risks associated with taking blood. It can be uncomfortable and result in fainting, localised pain, or bruising.

6. ***Are there any benefits in taking part?***

There are significant benefits from taking part in this research. Your participation could lead to improvements in your resilience and mental wellbeing and your participation will help us in evaluating the resilience intervention, which will guide improvements to the course before it is made nationally available.

7. ***Expenses and payments***

You will receive £30 for participation at the end of the study.

8. ***What will happen to any samples I give?***

Your saliva samples will be analysed for levels of a stress hormone called cortisol and destroyed immediately following analysis by incineration. We will process your blood samples within 24 hours of collection. Serum, which contains no cellular constituents, will be stored and analysed for an immune marker called C-reactive protein. Serum samples will be destroyed by incineration at the end of the study.

9. ***What happens to the data provided?***

Your name will be removed from your questionnaires and the anonymised research data will be stored on a password protected computer at the Oxford Centre for Anxiety Disorders and Trauma, Department of Experimental Psychology, University of Oxford. Anonymised saliva samples identified by a unique code will be analysed by the Biochemistry and Physiology Laboratory at the University of Surrey and immediately destroyed.

The Biochemistry and Physiology Laboratory at the University of Surrey will also process anonymised blood samples within 24 hours of collection, which will be analysed for a marker of inflammation called C-reactive protein. No cellular constituents will be saved or stored.

All information you provide will be strictly confidential. However, responsible members of the University of Oxford or King's College London may be given access to data for monitoring and/or audit or to suggest that specific analyses are carried out at the end of the study. We are collaborating with an expert in biological stress responses (Professor Carmine Pariante, King's College London), an expert in immune function (Professor Andrea Danese, King's College London) and an expert in health economics (Apostolos

For peer review only

Tsiachristas, University of Oxford). Our collaborators may suggest that we conduct specific statistical analyses at the end of the study.

In order to support transparency in research, some journals request that the aggregated anonymised data collected during a study are deposited within the UK data archive. If the journal with which we publish requires this, then the anonymised data would be submitted to the repository one year after the study is completed. Please be assured that only numerical data relating to the study outcomes (no personal identifying information whatsoever) would be held in the repository. We may also share the aggregated anonymised data with responsible researchers with an interest in resilience interventions.

To access the online courses, you will need to enter your unique code. Your responses in the modules you complete online will be linked to this code and stored anonymously on a password-protected database only accessible by the Principal Investigator and the research assistant. The responses linked to each code may be analysed.

Personal data relating to gender and ethnicity will be coded and then anonymised by linking it to your participant code rather than your name. Data will be stored on a password protected database on the Principal Investigator's computer. Your consent form will be stored in a locked filing cabinet in the Principal Investigator's office for 7 years and then destroyed.

10. *Will the research be published?*

The results from this study may be published within the next 7 years. You will not be personally identified in any literature and can obtain a copy of any publications from the contact numbers below.

11. *Who has reviewed this study?*

This study has been reviewed by, and received ethics clearance through, the University of Oxford Central University Research Ethics Committee.

12. *Who do I contact if I have a concern about the study or I wish to complain?*

If you have a concern about any aspect of this study, please speak to the Gabriella Tyson [01865 618 610] or Dr Jennifer Wild [01865 618 612], who will do their best to answer your query. The researcher should acknowledge your concern within 10 working days and give you an indication of how they intend to deal with it. If you remain unhappy or wish to make a formal complaint, please contact the relevant chair of the Research Ethics Committee at the University of Oxford who will seek to resolve the matter in a reasonably expeditious manner:

Chair, **Medical Sciences Inter-Divisional Research Ethics Committee**; Email: ethics@medsci.ox.ac.uk;
Address: Research Services, University of Oxford, Wellington Square, Oxford OX1 2JD

13. *Further Information and Contact Details*

If you would like to discuss the research with someone beforehand (or if you have questions afterwards), please contact:

Dr Jennifer Wild
Oxford Centre for Anxiety Disorders and Trauma
The Old Rectory
Paradise Square

For peer review only - http://bmjopen.bmj.com/site/about/guidelines.xhtml

Oxford
OX1 1TW
Tel: 01865 618 612
Email: Jennifer.wild@psy.ox.ac.uk

For peer review only

For peer review only - http://bmjopen.bmj.com/site/about/guidelines.xhtml

Oxford Centre for Anxiety Disorders and Trauma
The Old Rectory
Paradise Square, Oxford
OX1 1TW

UNIVERSITY OF OXFORD

Dr Jennifer Wild
Jennifer.wild@psy.ox.ac.uk Tel: 01865 618 612
Gabriella Tyson (Research Assistant)
Oxford telephone number: 01865 618 610
Oxford email address: gabriella.tyson@psy.ox.ac.ukx.ac.uk Tel: 01865 618 612

For peer review only

PARTICIPANT CONSENT FORM

CUREC Approval Reference:

A Study of Resilience Training for Student Paramedics

Purpose of Study: To evaluate a new resilience intervention developed for student paramedics

Please initial each box

1	I confirm that I have read and understand the information sheet version_____dated ________________ for the above study. I have had the opportunity to consider the information, ask questions and have had these answered satisfactorily.	☐
2	I understand that my participation is voluntary and that I am free to withdraw at any time, without giving any reason, and without any adverse consequences or academic penalty.	☐
3	I understand that research data collected during the study may be looked at by designated individuals from the University of Oxford where it is relevant to my taking part in this study. I give permission for these individuals to access my data.	☐
4	I understand that this project has been reviewed by, and received ethics clearance through, the University of Oxford Central University Research Ethics Committee.	☐
5	I understand who will have access to personal data provided, how the data will be stored and what will happen to the data at the end of the project.	☐
6	I give permission for the aggregated anonymised data to be shared with the UK data archive and other responsible researchers.	☐
7.	I consent to my telephone interviews with the research assistant being audio-recorded for quality assurance purposes.	☐
8	I understand how this research will be written up and published.	☐
9	I understand how to raise a concern or make a complaint.	☐
**10**	I understand that a blood and saliva will be taken during the study and that these samples will be tested for C-reactive protein and cortisol respectively. I understand that the samples will be destroyed after completion of this test or if I withdraw my consent for the test.	☐

11 I consider these samples a gift to the University of Oxford and I understand I will not gain any direct personal benefit from this. ☐

12 I agree to take part in the above study. ☐

Name of Participant	Date	Signature
Name of person taking consent	Date	Signature

For peer review only
